# Prevalence of cognitive frailty, reversible and potentially reversible cognitive frailty among older adults without dementia: a systematic review and meta-analysis

**DOI:** 10.1093/geronb/gbaf228

**Published:** 2025-11-09

**Authors:** Jinwei Bian, Zi Chen, Ying Gao, Fung Yee Lau, Daniel Yee Tak Fong, Edmond Pui Hang Choi, Pui Hing Chau

**Affiliations:** School of Nursing, The University of Hong Kong, Hong Kong, China; School of Nursing, The University of Hong Kong, Hong Kong, China; School of Nursing, The University of Hong Kong, Hong Kong, China; School of Nursing, The University of Hong Kong, Hong Kong, China; School of Nursing, The University of Hong Kong, Hong Kong, China; School of Nursing, The University of Hong Kong, Hong Kong, China; School of Nursing, The University of Hong Kong, Hong Kong, China

**Keywords:** Physical frailty, Cognitive impairment, Aging population

## Abstract

**Objectives:**

Cognitive frailty (CF) is characterized by the coexistence of physical frailty and cognitive impairment, while potentially reversible cognitive frailty (PRCF) and reversible cognitive frailty (RCF) refer to two related conditions that may be modifiable or responsive to interventions. This study aimed to estimate the pooled prevalence of CF, PRCF, and RCF among older adults without dementia.

**Methods:**

A search of six literature databases was conducted from inception to August 15, 2024. Cohort and cross-sectional studies reporting CF, PRCF, or RCF prevalence in older adults aged 60+ without dementia were included. Random-effects meta-analyses with logit-transformed prevalence were performed, along with subgroup analyses and meta-regression.

**Results:**

Of the 13,100 articles identified, 90 studies from 17 countries were eligible, with 63 studies on CF, 50 studies on PRCF, and 10 studies on RCF. In community settings, the pooled prevalence of CF and PRCF was 5% (95% confidence interval [CI]: 4%–6%) and 17% (95% CI: 13%–21%), respectively; in hospitals, 13% (95% CI: 8%–20%) and 32% (95% CI: 23%–42%); and in nursing homes, 22% (95% CI: 17%–29%) and 32% (95% CI: 1%–99%). The pooled prevalence of RCF in community settings was 21% (95% CI: 15%–29%). Studies with older participants showed higher CF prevalence. Moreover, studies with suboptimal sample sizes reported higher PRCF prevalence.

**Discussion:**

The prevalence of CF, PRCF, and RCF among older adults varies across settings, with PRCF and RCF showing notable rates. Further studies in underrepresented regions, along with age-stratified analyses, prior sample size calculations, and appropriate assessment tool selection, are needed.

Cognitive frailty (CF), proposed by the International Academy of Nutrition and Aging (IANA) and the International Association of Gerontology and Geriatrics (IAGG) in 2013, has gained significant attention as a public health concern, especially in the context of an aging global population (Kelaiditi et al., 2013). It refers to a heterogeneous clinical manifestation characterized by the coexistence of physical frailty and cognitive impairment (clinical dementia rating [CDR] = 0.5), excluding concurrent Alzheimer’s disease or other dementias (Kelaiditi et al., 2013). A strong association between CF, falls, disability, dementia, hospitalization, and mortality in older adults was reported (Bu et al., 2021; Zhang et al., 2022b). To facilitate public health strategies to prevent and tackle this condition, estimates of its prevalence were critical.

Despite its growing recognition, estimates of CF prevalence varied considerably across studies and populations due to ­differences in definitions, assessment tools, and demographic factors. To date, two meta-analyses have examined CF prevalence (Qiu et al., 2022; Zhang et al., 2022a). One meta-analysis of 24 studies reported a CF prevalence of 9% (95% confidence interval [CI]: 8%–11%) among community-dwelling older adults without dementia (Qiu et al., 2022). Another meta-analysis of 29 studies showed a CF prevalence of 6% (95% CI: 5%–7%) among community-dwelling older adults (Zhang et al., 2022a). However, these reviews may overestimate the CF prevalence by including studies with broader CF definitions or with populations that include those with dementia. The latter meta-analysis was also restricted to studies using the Fried frailty phenotype (FFP), limiting comparisons with other commonly used measures. Additionally, both reviews focused solely on community settings, leaving a knowledge gap in other settings like hospitals or nursing homes.

In an attempt to refine the framework for the definition and potential mechanisms of CF, Ruan et al. (2015) proposed two concepts: potentially reversible cognitive frailty (PRCF) and reversible cognitive frailty (RCF). Both involve either pre-physical frailty or physical frailty but differ in cognitive status: PRCF is characterized by mild cognitive impairment (MCI) and overlaps with the definition of CF proposed by IANA/IAGG, whereas RCF is characterized by subjective cognitive decline (SCD) and/or positive biomarkers. The definitions of PRCF and RCF by Ruan et al. are shown in Figure S1 (see online supplementary material). Prior research has shown that both PRCF and RCF were associated with dementia and mortality (Solfrizzi et al., 2017a, 2017b). Further studies showed that PRCF was significantly associated with a higher risk of functional disability and poor quality of life (Feng et al., 2017; Roppolo et al., 2017). Two meta-analyses on the prevalence of PRCF are currently available (Zhang et al., 2022a, 2024b). One meta-analysis of 24 studies reported a 12.2% prevalence of PRCF among community-dwelling older adults (Zhang et al., 2024b). However, this meta-analysis included studies using both the IANA/IAGG definition for CF and Ruan et al.’s definition for PRCF, which likely led to an underestimation of the pooled PRCF prevalence. Another meta-analysis of 14 studies reported a 16% (95% CI: 13%–19%) prevalence of PRCF among community-dwelling older adults (Zhang et al., 2022a). However, the included studies varied in baseline populations regarding the inclusion of individuals with dementia. Similar to CF, the pooled prevalence of PRCF among older adults in hospitals and nursing homes has not been estimated. Additionally, the pooled prevalence of RCF has yet to be investigated in any setting.

Understanding the current epidemiology of CF, PRCF, and RCF would be helpful for clinicians and researchers to further pursue relevant research and contribute to the government in making relevant public health strategic decisions. Therefore, this study aimed to systematically review existing literature reporting the prevalence of CF, PRCF, and RCF, to estimate the corresponding pooled prevalence based on standard definitions among well-defined populations in various settings, and to evaluate the impact of different factors on prevalence rates.

## Method

This systematic review and meta-analysis was conducted in accordance with the Preferred Reporting Items for Systematic Review and Meta-Analyses (PRISMA) guideline (Table S1, see online supplementary material) (Moher et al., 2009). The study was registered in PROSPERO under registration number CRD42024610759.

### Search strategy

In this systematic review and meta-analysis, two authors (J.B. and F.L.) independently searched PubMed, Cochrane Library, Embase, Web of Science, and two Chinese bibliographic databases (CNKI and Wanfang) from database inception to August 15, 2024, using free text terms and subject terms. In addition, the references of all eligible studies and related systematic reviews on similar topics were also searched to identify additional relevant studies. No language restriction was applied to any of the searches. Articles in languages other than English or Chinese were translated using the DeepL translator, with additional guidance sought from translation experts to ensure accuracy. The detailed search strategies are in Table S2 (see online supplementary material).

### Selection criteria

Studies were considered eligible if they met the following criteria: (1) participants being general older people aged 60 years and above; (2) participants recruited from the community, hospital, or nursing home settings; (3) cohort or cross-sectional study designs; (4) randomly recruited participants; (5) participants excluded for any type of dementia, as defined by report of dementia or validated cognitive test performance meeting cutoffs for dementia; (6) with reported prevalence or reported sufficient data for calculation of prevalence; (7) physical frailty and cognitive status, that were used to define CF, PRCF, and RCF, based on validated assessment methods. CF was defined as physical frailty plus MCI. PRCF was defined as pre-physical frailty or physical frailty plus MCI. RCF was defined as pre-physical frailty or physical frailty plus SCD. While the original definition by Ruan et al. (2015) included positive biomarkers for RCF, such measures are rarely available in large epidemiological studies. Given our focus on population-based prevalence estimates, only the SCD-based definition of RCF was adopted. Studies were excluded if they: (1) were review articles, book chapters, case reports, protocols, conference abstracts, letters, comments, short communications, posters, and reports; (2) were case-control studies or randomized controlled trials; (3) were targeting participants of particular disease group or health conditions (such as multi-morbidity); (4) did not have full text articles. When more than one identified record reported the prevalence of the same condition in the same population, only the study using the most common definition was selected. If the studies used the same definition, only the study with the largest sample was selected. If the sample sizes were the same, only the latest study was included. Studies reporting prevalence for two or more distinct cohorts within one article were treated as separate studies.

### Study selection and data extraction

All articles identified were imported into EndNote 21, and duplicates were removed. Two authors (J.B. and F.L.) independently evaluated eligibility, and discrepancies were resolved through discussion with a senior author (P.C.). We also contacted the corresponding authors to obtain the required information if it was not reported.

Data from each of the eligible studies were extracted using a piloted template. Data were extracted by two independent reviewers (J.B. and Y.G.). The following data were extracted: (1) citation; (2) study details (region, survey period, setting, data source, study design, and sample size); (3) participant information (female proportion and mean age); (4) assessment methods; (5) prevalence. Prevalence was calculated based on the available data if it was not directly provided. Baseline data were extracted for the cohort study.

### Risk of bias evaluation

We assessed the risk of bias for the included studies using the Joanna Briggs Institute Critical Appraisal Checklist for Prevalence Studies (Munn et al., 2020, 2014). This checklist consists of nine criteria, including sample frame, sampling method, sample size, study subjects and setting, data analysis, validity of methods, condition measurement, statistical analysis, and response rate. We evaluated the criteria separately for each outcome when a study reported more than one outcome of CF, PRCF, and RCF. Criteria 3, “Was the sample size adequate?,” was determined by assessing whether the sample size met the requirement for estimating the prevalence with 5% precision,based on the formula $n=\frac{z^{2}p(1-p)}{d^{2}}$, where z=1.96,d=0.05,and p was the prevalence reported by each included study (Munn et al., 2020). Studies with a score up to 49% reporting “yes” indicated a high risk of bias, 50%–69% indicated a moderate risk of bias, and a score of 70% or higher reporting “yes” indicated a low risk of bias (Valesan et al., 2021). Risk of bias assessment was completed by two independent reviewers (J.B. and Z.C.) and any discrepancy were resolved by a senior researcher (P.C.).

### Data analysis

The pooled prevalence and corresponding 95% CIs were calculated using a random-effects model with logit transformation (Stijnen et al., 2010). The random-effects model was selected a priori due to the expected heterogeneity among the included studies. Heterogeneity was assessed using Cochran’s Q and $I^{2}$ statistics, with $I^{2}$ values of 25%, 50%, and 75% indicating low, moderate, and high heterogeneity, respectively (Higgins et al., 2003). Publication bias was evaluated by funnel plots and Egger’s test for funnel plot asymmetry.

Potential sources of heterogeneity were investigated using subgroup analyses and meta-regression. Subgroup analyses were performed based on categorical variables, including setting, region, study design, survey period, sample size, and assessment methods. Subgroup differences were assessed using the $\chi^{2}$ test. In the subgroup analyses, sample size was dichotomized as meeting or not meeting the sample size requirement, consistent with criteria 3 in the risk of bias assessment. The survey period was categorized into the last decade (2015–2024) and earlier (pre-2015). For studies spanning multiple years, the midpoint of the survey period was used for classification. Meta-regression models with logit transformation were also fitted to further explore heterogeneity across study findings. Variables included in the final multivariable analyses were selected using a stepwise selection method. For studies reporting age-specific prevalence, we conducted a narrative synthesis. We also conducted a multicollinearity analysis and used a variance inflation factor >4 to identify the presence of multicollinearity, and no multicollinearity was found in all multivariable analyses (Mason, 1987).

We conducted three sensitivity analyses to assess the robustness of the results. First, leave-one-out influence analyses were conducted to recalculating the pooled effect estimate iteratively by omitting one study at a time. Second, the Baujat plots were plotted with the included studies, and studies contributing more than 200 to heterogeneity were excluded. A Baujat plot illustrated the influence of each study on the overall effect (Baujat et al., 2002). Third, studies with high risk of bias were excluded.

Our analysis was performed with RStudio using the R package meta and metafor (Schwarzer et al., 2015). Statistical significance was set at a two-sided *p*-value of less than .05.

## Results

### Included studies and demographics

**Figure 1.**
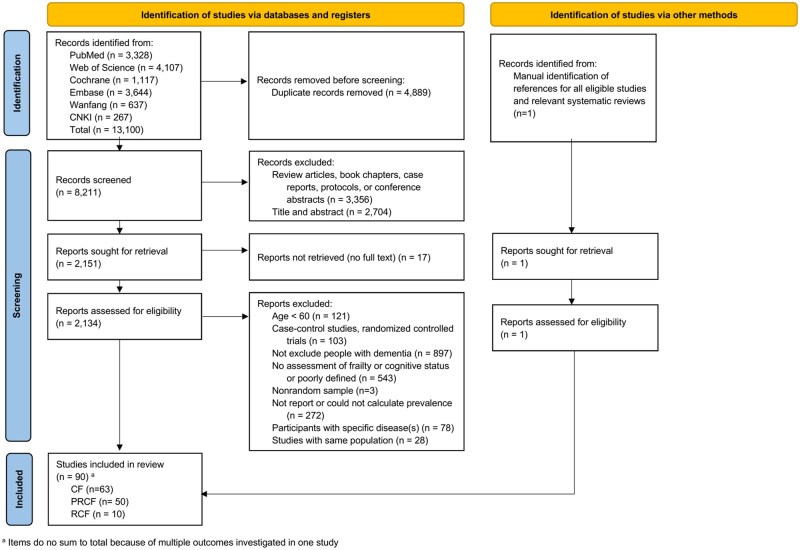
PRISMA flow diagram of study selection. CF = cognitive frailty; PRCF = potentially reversible cognitive frailty; RCF = reversible cognitive frailty.

Of 13,100 records identified, 8,211 were screened and 2,134 articles were reviewed for eligibility. Finally, 90 studies from 17 countries were found to be eligible for analysis, including 63 studies for CF, 50 studies for PRCF and 10 studies for RCF (Figure 1 and Table S3, see online supplementary material).

Among the 90 included studies, 69 were written in English and 21 in Chinese; 75 were cross-sectional studies and 15 were cohort studies; 66 studies were conducted in community settings, 18 in hospitals, and six in nursing homes. Geographically, 74 studies were conducted in Asia, nine in Europe, and seven in the Americas, with most originating from China (*n *= 47), Japan (*n *= 9), and South Korea (*n *= 6). All included studies were published between 2009 and 2024, with sample sizes ranging from 43 to 39,148. The majority of the samples were female, with 70 studies reporting a female proportion greater than 50%, ranging from 24.7% to 80.2%. The minimum recruitment age varied from 60 to 90 years, with the mean age ranging from 66.0 to 93.6 years.

The included studies employed various assessment methods (Table S4, see online supplementary material). For physical function assessment, 50 studies used FFP, 26 used the FRAIL scale, four used measures of slow walking speed and/or muscle weakness, three used the Frailty Index, two used the Clinical Frailty Scale, two used the Kihon Checklist, and one each used the Study of Osteoporotic Fractures Index, the Timed Up and Go test, and the Tilburg Frailty Index. For cognitive function assessment, 43 studies used Mini-Mental State Examination (MMSE), 11 used Montreal Cognitive Assessment (MoCA), six used CDR, three used National Center for Geriatrics and the Gerontology-Functional Assessment Tool (NCGG-FAT), two used Mini-Cog, and one each used validated scales including Rowland Universal Dementia Assessment Scale, Ascertain Dementia-8, Hasegawa Dementia Scale, Korean Dementia Screening Questionnaires-Prescreening, Repeatable Battery for the Assessment of Neuropsychological Status, Rapid Cognitive Screen, self-administered dementia checklist, Short Portable Mental Status Questionnaire, immediate and delayed recalls, and 16 used multiple cognitive tests. For dementia assessment, 18 studies used cognitive tests, 62 used non-cognitive tests (such as diagnosis of dementia and history of dementia), and 10 used a combination of cognitive and non-cognitive tests. Among the 10 studies on RCF, six assessed SCD through self-reported memory complaints, two used the simplified SCD questionnaire, and one each used the Everyday Memory Questionnaire and SCD questionnaire MyCog scores.

### Risk of bias assessment

The risk of bias of the included studies is described in Figure S2 (see online supplementary material). Only two were rated as high risk of bias, mainly due to suboptimal sample size and lack of detailed descriptions of random sampling methods and response rates.

### Prevalence of CF

**Figure 2.**
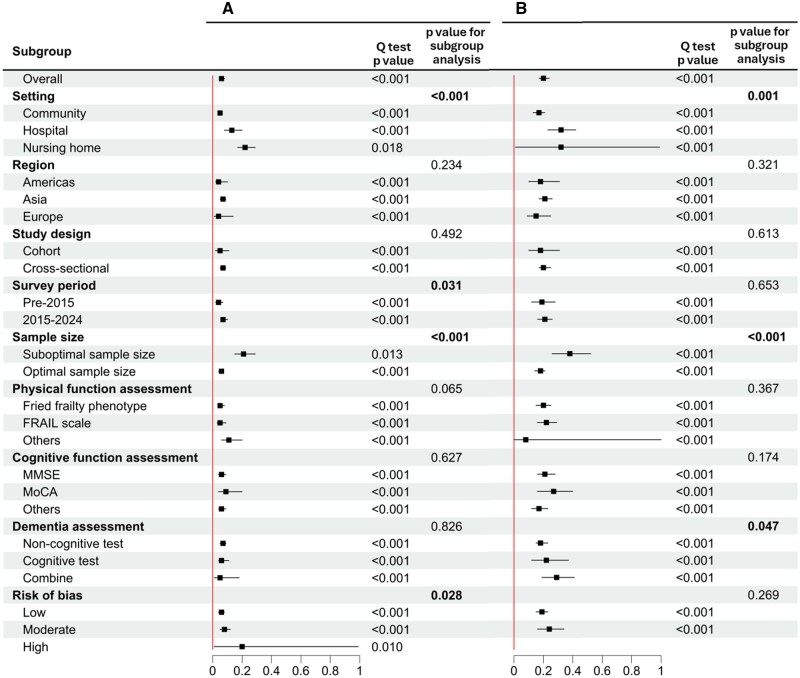
Subgroup analyses for the prevalence of (A) CF and (B) PRCF. See Figure S3 (see online supplementary material) for the expanded version of the figure. CF = cognitive frailty; MMSE = Mini-Mental State Examination; MoCA = Montreal Cognitive Assessment; PRCF = potentially reversible cognitive frailty.

The pooled prevalence of CF was 6% (95% CI: 5%–8%, *p *< .001) among older adults without dementia. Subgroup analyses showed a CF prevalence of 5% (95% CI: 4%–6%, *p *< .001) in the community, compared to 13% (95% CI: 8%–20%, *p *< .001) in hospitals and 22% (95% CI: 17%–29%, *p *= .018) in nursing homes (*p *< .001 for subgroup differences). The prevalence of CF was 4% (95% CI: 2%–7%, *p *< .001) in pre-2015, compared to 7% (95% CI: 6%–10%) in 2015–2024 (*p *= .031 for subgroup differences). Significant differences were also observed in sample size, with a prevalence of 21% (95% CI: 15%–29%, *p *= .013) for samples not meeting the optimal sample size and 6% (95% CI: 4%–7%, *p *< .001) for samples meeting the optimal sample size (*p *< .001 for subgroup differences). Regarding risk of bias, the prevalence of CF was 6% (95% CI: 4%–8%, *p *< .001) for low risk of bias, 8% (95% CI: 5%–12%, *p *< .001) for moderate risk of bias, and 20% (95% CI: 1%–99%, *p *= .010) for high risk of bias (*p *= .028 for subgroup differences) (Figure 2A).

Significant heterogeneity was observed across studies ($I^{2}$ = 98.8%; *p *< .001). In the univariable meta-regression (Table 1), settings (*p *< .001), survey period (*p *= .020), sample size (*p *= .001), mean age (*p *= .001), and physical function assessments (*p *= .042) were significantly associated with heterogeneity. The multivariable regression model, developed using the stepwise selection method, explained 34.0% of the total variance (Table 1). The prevalence of CF was 2.15 times (95% CI: 1.08–4.28, *p *= .030) in hospitals than in the community. Additionally, studies with older participants showed higher CF prevalence (*p *= .015).

**Table 1.** The results of the meta-regression of the prevalence of cognitive frailty.

Variables	Univariable analysis		Multivariable analysis^d^	
	*B* (95% CI)	*p^e^*	*B* (95% CI)	*p^e^*
**Setting (ref: community)**		**<.001**		**.042**
** Hospital**	2.98 (1.61, 5.53)	**.001**	2.15 (1.08, 4.28)	**.030**
** Nursing home**	5.85 (2.49, 13.72)	**<.001**	2.15 (0.76, 6.09)	.150
**Region (ref: Asia)**				
** Non-Asia**	0.59 (0.30, 1.15)	.121		
**Study design (ref: cohort)**				
** Cross-sectional**	1.29 (0.67, 2.50)	.445		
**Survey period (ref: pre-2015)^a^**				
** 2015-2024**	1.96 (1.11, 3.46)	**.020**	1.51 (0.89, 2.55)	.125
**Sample size (ref: optimal)**				
** Suboptimal**	4.42 (1.87, 10.40)	**.001**	2.34 (0.85, 6.43)	.099
**Mean age^b^**	1.10 (1.04, 1.15)	**.001**	1.07 (1.01, 1.13)	**.015**
**Female proportion^c^**	1.38 (0.06, 30.09)	.837		
**Physical frailty (ref: Fried Frailty phenotype)**		**.042**		
** FRAIL scale**	0.96 (0.49, 1.85)	.892		
** Others**	2.18 (1.15, 4.13)	**.017**		
**Cognitive impairment (ref: MMSE)**		.560		
** MoCA**	1.47 (0.63, 3.44)	.376		
** Others**	0.91 (0.51, 1.61)	.744		
**Dementia (ref: non-cognitive test)**		.731		
** Cognitive test**	1.00 (0.52, 1.91)	.990		
** Combine**	0.69 (0.28, 1.74)	.436		
**Risk of bias (ref: low risk of bias)**		.119		
** Moderate risk of bias**	1.47 (0.80, 2.70)	.210		
** High risk of bias**	4.10 (0.85, 19.75)	.079		

*Note*. MMSE = Mini-Mental State Examination; MoCA = Montreal Cognitive Assessment; ref = reference.

a Only 59 studies were included in the univariable analysis due to missing data on the survey period.

b Only 52 studies were included in the univariable analysis due to missing data on mean age.

c Only 62 studies were included in the univariable analysis due to missing data on female proportion.

d Only 49 studies were included in multivariable analysis due to missing data on survey period and mean age.

e Values in bold indicate statistically significant differences at the 0.05 level (p < .050).

### Prevalence of PRCF

The pooled prevalence of PRCF was 20% (95% CI: 17%–24%, *p *< .001) among older adults without dementia. Subgroup analyses showed a PRCF prevalence of 17% (95% CI: 13%–21%, *p *< .001) in the community, compared to 32% (95% CI: 23%–42%, *p *< .001) in hospitals and 32% (95% CI: 1%–99%, *p *< .001) in nursing homes (*p *= .001 for subgroup differences). Significant differences were also observed in sample size, with a prevalence of 38% (95% CI: 26%–52%, *p *< .001) for samples not meeting the optimal sample size and 18% (95% CI: 14%–21%, *p *< .001) for samples meeting the optimal sample size (*p *< .001 for subgroup differences). Regarding dementia assessments, the pooled prevalence of PRCF was 18% (95% CI: 15%–23%, *p *< .001) for non-cognitive tests, 22% (95% CI: 12%–37%, *p *< 0.001) for cognitive tests, and 29% (95% CI: 19%–41%, *p *< .001) for the combination of cognitive and non-cognitive tests (*p *= .047 for subgroup differences) (Figure 2B).

Significant heterogeneity was observed across studies ($I^{2}$ = 98.8%; *p *< .001). In the univariable meta-regression (Table 2), settings (*p *= .010) and sample size (*p *< .001) were significantly associated with heterogeneity. Based on the stepwise selection method, a multivariable regression model was developed, which explained 43.5% of the variance (Table 2). The prevalence of PRCF was 2.48 times (95% CI: 1.43–4.29, *p *= .001) in hospitals and 3.61 times (95% CI: 1.22–10.66, *p *= .020) in nursing homes than in the community. Studies with suboptimal sample sizes had higher PRCF prevalence than those with optimal sample sizes (*p *= .003).

**Table 2.** The results of the meta-regression of the prevalence of PRCF.

Variables	Univariable analysis		Multivariable analysis^c^	
	B (95% CI)	*p^d^*	B (95% CI)	*p^d^*
**Setting (ref: community)**		**.010**		**.001**
** Hospital**	2.32 (1.39, 3.85)	**.001**	2.48 (1.43, 4.29)	**.001**
** Nursing home**	2.29 (0.79, 6.60)	.126	3.61 (1.22, 10.66)	**.020**
**Region (ref: Asia)**				
** Non-Asia**	0.73 (0.42, 1.27)	.263		
**Study design (ref: cohort)**				
** Cross-sectional**	1.18 (0.61, 2.29)	.616		
**Survey period (ref: pre-2015)^a^**				
** 2015-2024**	1.14 (0.67, 1.94)	.620		
**Sample size (ref: optimal)**				
** Suboptimal**	2.89 (1.63, 5.12)	**<.001**	2.63 (1.39, 4.98)	**.003**
**Mean age^b^**	1.06 (0.99, 1.14)	.109	1.00 (0.93, 1.06)	.773
**Female proportion**	0.37 (0.05, 2.98)	.351		
**Physical frailty (ref: Fried Frailty phenotype)**		.201		
** FRAIL scale**	1.14 (0.71, 1.84)	.577		
** Others**	0.38 (0.11, 1.25)	.111		
**Cognitive impairment (ref: MMSE)**		.235		
** MoCA**	1.35 (0.68, 2.70)	.395		
** Others**	0.75 (0.46, 1.23)	.260		
**Dementia (ref: non-cognitive test)**		.284		.125
** Cognitive test**	1.26 (0.69, 2.30)	.452	1.62 (0.88, 2.96)	.120
** Combine**	1.81 (0.83, 3.91)	.135	1.79 (0.91, 3.54)	.093
**Risk of bias (ref: low risk of bias)**				
** Moderate risk of bias**	1.35 (0.83, 2.22)	.229		

*Note*. MMSE = Mini-Mental State Examination; MoCA = Montreal Cognitive Assessment; PRCF = potentially reversible cognitive frailty; ref = reference.

a Only 49 studies were included in the univariable analysis due to missing data on the survey period.

b Only 40 studies were included in the univariable analysis due to missing data on mean age.

c Only 40 studies were included in the multivariable analysis due to missing data on mean age.

d Values in bold indicate statistically significant differences at the 0.05 level (p < .050).

### Prevalence of RCF

**Figure 3.**
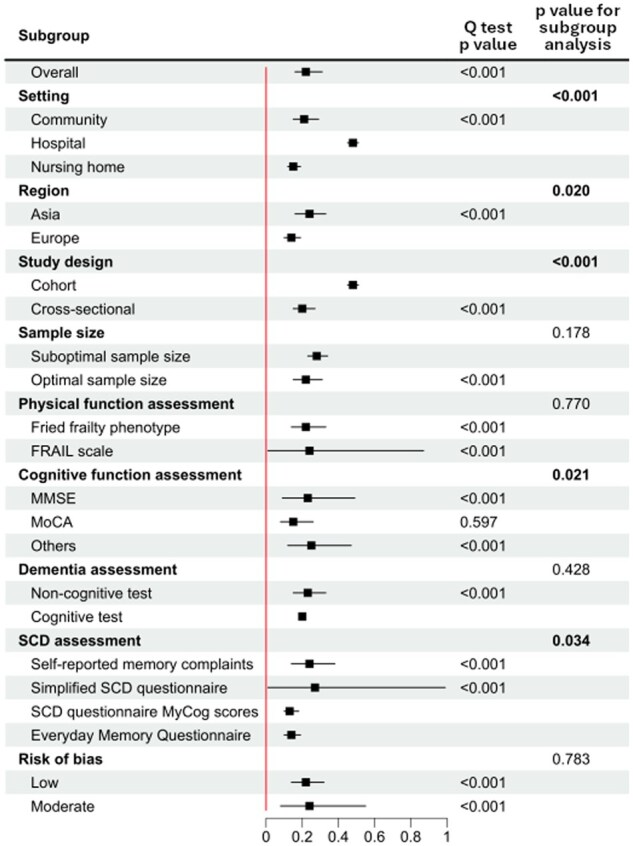
Subgroup analyses for the prevalence of RCF. See Figure S3 (see online supplementary material) for the expanded version of the figure. MMSE = Mini-Mental State Examination; MoCA = Montreal Cognitive Assessment; SCD = subjective cognitive decline.

The pooled prevalence of RCF was 22% (95% CI: 16%–31%, *p *< .001) among older adults without dementia. The prevalence of RCF in the community was 21% (95% CI: 15%–29%, *p *< .001), while only one study each from hospitals and nursing homes was included, reporting prevalence rates of 48% (95% CI: 45%–51%) and 15% (95% CI: 12%–19%), respectively. Subgroup analyses also showed that the prevalence of RCF was 24% (95% CI: 16%–33%, *p *< .001) in Asia, compared to 14% (95% CI: 10%–19%) in Europe (*p *= .020 for subgroup differences). It was 48% (95% CI: 45%–51%) for cohort studies and 20% (95% CI: 15%–27%, *p *< .001) for cross-sectional studies (*p *< .001 for subgroup differences). Significant differences were also observed in cognitive function assessments, which were 23% (95% CI: 9%–49%, *p *< .001) for MMSE, 15% (95% CI: 8%–26%, *p *= .597) for MoCA, and 25% (95% CI: 12%–47%, *p *< .001) for others (*p *= .021 for subgroup differences). Regarding SCD assessments, the pooled prevalence of RCF was 24% (95% CI: 14%–38%, *p *< .001) for self-reported memory complaints, 27% (95% CI: 1%–99%, *p *< .001) for simplified SCD questionnaire, 14% (95% CI: 10%–19%) for the Everyday Memory Questionnaire, and 13% (95% CI: 10%–18%) for SCD questionnaire MyCog scores (*p *= .034 for subgroup differences) (Figure 3).

Significant heterogeneity was observed across studies ($I^{2\;}$ = 98.2%; *p *< .001). Due to the limited number of included studies on RCF, only univariable meta-regression analyses were conducted, and no variables were found to be statistically significant (Table 3).

**Table 3.** The results of the meta-regression of the prevalence of RCF.

Variables	Univariable analysis ^a^ ^b^ ^c^	
	*B* (95% CI)	*p*
**Setting (ref: community)**		
** Hospital/nursing home**	1.58 (0.61, 4.13)	.349
**Mean age**	0.98 (0.86, 1.11)	.728
**Female proportion**	5.85 (0.09, 362.49)	.402
**Physical frailty (ref: Fried Frailty phenotype)**		
** FRAIL scale**	1.10 (0.40, 3.00)	.856
**Cognitive impairment (ref: MMSE)**		.237
** MoCA**	0.57 (0.18, 1.78)	.296
** Others**	1.13 (0.46, 2.77)	.794
**SCD (ref: Self-reported memory complaints)**		
** Others**	0.79 (0.35, 1.81)	.592
**Risk of bias (ref: low risk of bias)**		
** Moderate risk of bias**	1.16 (0.51, 2.66)	.718

*Note*. MMSE = Mini-Mental State Examination; MoCA = Montreal Cognitive Assessment; RCF = reversible cognitive frailty; ref = reference; SCD = subjective cognitive decline.

a Since all included studies were conducted after 2015, meta-regression on survey period was not performed.

b Due to the limited number of included studies on reversible cognitive frailty, only univariable meta-regression analysis was performed.

c Meta-regression was not conducted for variables such as region, study design, sample size, and dementia assessment methods, as some subgroups were represented by only a single study.

### Age-specific prevalence

28 studies reported age-specific prevalence, including 16 on CF, 12 on PRCF, and three on RCF. In community settings, CF prevalence ranged from 0.8% to 15.5% at 60–74 years and 2.5%–32.8% at ≥75 years, with one study reporting 50.1% at ≥90 years. PRCF prevalence ranged from 2%–17.2% at 60–69 years, 5.1%–33.2% at 70–79 years, and 15.7%–74.5% at ≥80 years. RCF prevalence ranged from 15.6% to 38.4% at 60–69 years, 15.9%–39.2% at 70–79 years, and 8.6%–31.8% at ≥80 years. In hospital settings, CF prevalence ranged from 6.6% to 21.0% at 60–79 years and 37.5%–76.4% at ≥80 years, while in nursing homes, it ranged from 2.3% to 16.7% at 60–74 years and 25.0%–31.9% at ≥75 years. Age-specific PRCF prevalence was reported in only one study for each setting: in hospitals, it was 24.4% at 60–69 years, 35.0% at 70–79 years and 55.6% at ≥80 years (Li et al., 2024); in nursing homes, it was 15.8% at 60–69 years, 16.7% at 70–79 years and 18.9% at ≥80 years (Liu et al., 2022). No study reported age-specific RCF prevalence in hospitals or nursing homes.

### Sensitivity analysis

First, the leave-one-out sensitivity analysis revealed no significant changes in the reported prevalence when excluding each study individually (Figure S4, see online supplementary material). Second, Baujat plots were developed to identify studies contributing most to heterogeneity (Figure S4, see online supplementary material). After excluding seven CF studies and seven PRCF studies, the prevalence rates remained largely unchanged at 6% (95% CI: 5%–8%) for CF and 20% (95% CI: 16%–23%) for PRCF, with $I^{2}$ values of 97.5% and 97.8%, respectively. After excluding a highly heterogeneous RCF study conducted by Suprawesta et al. (Suprawesta et al., 2024), the pooled prevalence slightly reduced to 20% (95% CI: 15%–27%), with $I^{2}$ values of 97.0% (Figure S6, see online supplementary material). Third, the sensitivity analysis indicated that prevalence estimates were not affected by the exclusion of studies with a high risk of bias (Figure S7, see online supplementary material).

### Publication bias

The results of Egger’s test indicated no statistically significant differences in the prevalence of CF (*p *= .246), PRCF (*p *= .433), and RCF (*p *= .777), and the funnel plots were generally symmetrical (Figure S8, see online supplementary material). Therefore, it could be considered that publication bias was unlikely.

## Discussion

In this systematic review and meta-analysis, we identified the results of 90 studies involving 180,932 older adults without dementia. In the community, the pooled prevalence of CF and PRCF was 5% and 17%, respectively; in hospitals, 13% and 32%; and in nursing homes, 22% and 32%. The pooled prevalence of RCF in the community was 21%. In addition, sample size and mean age were found to be associated with prevalence.

In the community setting, our study reported a CF prevalence of 5% (95% CI: 4%–6%) among community-dwelling older adults without dementia, lower than the 9% (95% CI: 8%–11%) reported in a previous meta-analysis. The difference is possibly due to the latter’s inclusion of studies that used the PRCF definition to estimate CF (Qiu et al., 2022). Similarly, our study reported a higher PRCF prevalence (17%, 95% CI: 13%–21%) than the previous meta-analysis (12.2%), which may be attributed to the inclusion of studies that used the IANA/IAGG definition for CF to estimate PRCF (Zhang et al., 2024b). A 2022 meta-analysis simultaneously reported a CF prevalence of 6% (95% CI: 5%–7%) and a PRCF prevalence of 16% (95% CI: 13%–19%) (Zhang et al., 2022a), which is comparable to our estimates, despite being based solely on studies using FFP for physical assessment and involving different baseline populations (e.g., individuals without dementia vs. the general population). Regarding RCF, our review was the first to examine its prevalence among older adults without dementia, reporting a pooled RCF prevalence of 21% (95% CI: 15%–29%) in the community. This suggested that RCF was common among community-dwelling older adults.

Our study found that the prevalence of CF and PRCF was higher in hospitals than in the community, consistent with two previous reviews indicating that clinical-based studies report higher prevalence rates compared to community-based studies (Panza et al., 2018; Sugimoto et al., 2018). This may be due to the high prevalence of underlying health conditions in hospitalized older adults that require medical attention. Previous studies have found that cardiovascular disease and chronic kidney disease were associated with the onset and progression of CF (Ijaz et al., 2024; Yuan et al., 2024). Besides, a lower pooled prevalence of RCF observed in the sensitivity analysis after excluding the study by Suprawesta et al. (2024) may also be attributed to this reason. As a hospital-based cohort study, it included older adults with poorer health conditions, and its exclusion likely contributed to the observed decrease in the pooled prevalence.

This study is the first systematic review and meta-analysis to examine the prevalence of CF and PRCF in nursing homes, and the results revealed a higher prevalence of PRCF in nursing homes than in the community. This may stem from the fact that nursing home residents are typically older, have lower functional status, and often suffer from multiple chronic conditions, all of which are closely linked to physical and cognitive decline (Sugimoto et al., 2022; Tate et al., 2023). Regarding RCF, our analysis included only one study in nursing homes (Zhang et al., 2024a) and one in hospitals (Suprawesta et al., 2024), which prevented the calculation of pooled prevalence estimates. Further research in both settings is needed to provide more comprehensive and robust evidence on the prevalence of RCF.

Our study also revealed that an older mean age of participants was associated with a higher prevalence of CF, consistent with previous reviews (Liu et al., 2023; Zhang et al., 2022a). Age-related physiological changes in the brain and the increased burden of comorbidities were known to contribute to physical and cognitive decline, potentially explaining the increased prevalence of CF in older populations (Ma et al., 2019). This highlights the need for future research to incorporate age stratification or adjustment when estimating CF prevalence.

In addition, suboptimal sample sizes were related to higher prevalence estimates for PRCF, possibly because smaller sample sizes, especially those falling below optimal thresholds, were more susceptible to systematic error and sampling bias, which could lead to inflated prevalence estimates. Therefore, future studies should conduct prior sample size calculations, which are essential for accurate prevalence estimation.

Assessment methods may also contribute to variations in the reported prevalence. Regarding physical assessment, according to the univariable meta-regression, our study found that CF prevalence based on measurements other than FFP and the FRAIL scale was 2.18 times (95% CI: 1.15–4.13, *p *= .017) of that using FFP. This aligns with a prior review, which reported a CF prevalence of 9.8% (95% CI: 6.1%–13%) among community-dwelling older adults with diabetes when using FFP, compared to a higher prevalence of 19% (95% CI: 15%–23%) in studies that used other measurements (Lyu et al., 2023). Regarding dementia assessments, the subgroup analysis reported significant differences (*p *= .047) in the pooled PRCF prevalence among non-cognitive tests, cognitive tests, and the combination of cognitive and non-cognitive tests. This may be because, compared to non-cognitive tests, cognitive tests could identify undiagnosed dementia cases, particularly among rural residents who might lack a diagnosis of dementia due to limited access to medical care or low awareness, leading to varying PRCF prevalences. However, it was not significant after taking into account other factors in the multivariable analysis. Regarding SCD assessment, the subgroup analysis reported significant differences (*p *= .034) in the pooled RCF prevalence in our study. This may be due to two studies that used the Everyday Memory Questionnaire (14% prevalence) and the SCD Questionnaire MyCog scores (13% prevalence), respectively, as SCD assessment methods (Gaspar et al., 2022; Ruan et al., 2021). In contrast to studies measuring SCD through self-reported memory complaints (with a pooled RCF prevalence of 24%), these studies employed more objective criteria to diagnose SCD, possibly resulting in the lower prevalence. Therefore, future studies should further evaluate and compare various assessment methods to establish standardized criteria that can accurately capture the prevalence across different populations.

The strengths of this study lie first in its comprehensive literature search, which encompasses studies from various countries and regions across multiple databases, thereby enhancing the generalizability and representativeness of the findings. Second, our study clearly defined CF, PRCF, and RCF, and employed strict inclusion and exclusion criteria to minimize conceptual ambiguity and enhance the reliability of the results. Third, the study focused exclusively on older adults without dementia, avoiding potential bias from variations in baseline populations (inclusion of dementia or not). This approach provides a clear picture of the prevalence of CF, PRCF, and RCF before dementia, which could guide preventive strategies. Fourth, this review incorporated studies from diverse settings such as communities, hospitals, and nursing homes, enhancing the external validity of the results and allowing for a more nuanced understanding of the prevalence across various environments. Lastly, we employed advanced statistical approaches, including subgroup analyses and meta-regression, to gain insights into the factors affecting prevalence, thereby providing valuable guidance for future studies. Nonetheless, this study has some limitations. First, the number of epidemiological studies on CF, PRCF, and RCF is inadequate, particularly in certain regions (e.g., African countries) and settings (e.g., nursing homes and hospitals). Notably, regarding RCF prevalence, only 10 studies were available overall, with hospitals and nursing homes each represented by a single study, precluding pooled estimates. Future studies should focus on these underrepresented regions and settings to provide more comprehensive and representative prevalence estimates. Second, most included studies were conducted in Asian populations, which may limit the applicability of our findings. Ethnic and cultural differences can influence the development and presentation of frailty, as well as the validity and interpretation of cognitive assessments and healthcare utilization patterns. These factors, individually or combined, may affect prevalence estimates. Additional primary studies focusing on these underrepresented groups are needed in future research to enhance the generalizability of findings. Third, while we conducted a narrative synthesis of age-specific prevalence, the limited number of studies and the inconsistent age cutoffs precluded more comprehensive analyses. Future research should collect and report age-stratified prevalence to enable more robust comparisons across age groups and populations. Fourth, despite conducting subgroup and meta-regression analyses, substantial residual heterogeneity remained, possibly due to differences in study populations, assessment tools, and study designs. Such residual heterogen­eity may limit the generalizability of the pooled estimates. Last, given the exploratory nature of the subgroup analyses and the heterogeneity across studies, we did not apply formal corrections for multiple comparisons, which may increase the risk of false positive errors. Consequently, the findings—particularly those with borderline significance—should be interpreted with caution, and future confirmatory studies are warranted to validate these results.

Our systematic review also has important epidemiology implications. The high prevalence of PRCF and RCF reported in our study emphasizes the importance of addressing these reversible conditions alongside CF. Since both PRCF and RCF represent reversible states, early detection and timely intervention may improve impairment, prevent further cognitive and functional deterioration, and reduce the risk of progression to irreversible cognitive decline, particularly in aging communities. A 24-week randomized controlled trial among community-dwelling older adults with PRCF found that after 12 weeks of multi-domain intervention—including multicomponent exercise, cognitive stimulation, dietary counseling, and psychosocial support—74.1% of participants no longer met the PRCF criteria, with 63% maintaining this improvement at 24 weeks (Murukesu et al., 2024). The substantial PRCF and RCF prevalence in hospitals and nursing homes also underscores the need for cognitive and physical assessments in routine care to identify at-risk individuals and enable timely interventions, thereby improving overall patient health and reducing unnecessary long-term care needs. Additionally, by quantifying prevalence in different settings, the study underscores the need for targeted health policies and resource allocation. The study also highlights the role of adequate sample sizes and appropriate assessment tools in accurate prevalence estimation.

## Conclusion

The present study provides a comprehensive and up-to-date analysis of CF, PRCF, and RCF prevalence, highlighting the impact of settings, sample size, and age. Further research on CF, PRCF, and RCF is needed, particularly in underrepresented regions and settings, along with age-stratified analyses, prior sample size calculations, and appropriate assessment tools selection.

## Supplementary Material

gbaf228_Supplementary_Data

## Data Availability

The data supporting the findings of this study are derived from previously published studies. The study was registered in PROSPERO under registration number CRD42024610759.
