# Supplementary material for: Prevalence of cognitive frailty, reversible and potentially reversible cognitive frailty among older adults without dementia: a systematic review and meta-analysis
Source: J Gerontol B Psychol Sci Soc Sci. 2025 Nov 9;81(1):gbaf228. doi: 10.1093/geronb/gbaf228 (PMC12771507; doi:10.1093/geronb/gbaf228)
Supplement: gbaf228_Supplementary_Data [file gbaf228_supplementary_data.docx]

***The Journals of Gerontology, Series B: Psychological Sciences and Social Sciences* Supplementary Material: Bian, Chen, Gao, Lau, Fong, Choi, & Chau. Prevalence of cognitive frailty, reversible and potentially reversible cognitive frailty among older adults without dementia: a systematic review and meta-analysis.**

**Supplementary Table 1.** PRISMA 2020 Checklist

**Supplementary Table 2.** Search strategies

**Supplementary Table 3.** Characteristics of the included studies

**Supplementary Table 4.** Assessment tools used in the included studies

**Supplementary Figure 1.** Definitions of cognitive frailty by IANA/IAGG, as well as those for potentially reversible cognitive frailty and reversible cognitive frailty by Ruan et al

**Supplementary Figure 2.** Risk of bias assessment for the included studies for (A) CF, (B) PRCF, and (C) RCF

**Supplementary Figure 3.** Subgroup analyses for prevalence of (A) cognitive frailty, (B) potentially reversible cognitive frailty, and (C) reversible cognitive frailty (full versions of Figures 2 and 3 in the article).

**Supplementary Figure 4.** Sensitivity analysis using the Leave-one-out approach for (A) CF, (B) PRCF, and (C) RCF

**Supplementary Figure 5.** Baujat plots of (A) CF, (B) PRCF, and (C) RCF

**Supplementary Figure 6.** Prevalence of (A) CF, (B) PRCF, and (C) RCF after excluding studies with high heterogeneity contribution

**Supplementary Figure 7.** Prevalence of CF after excluding studies with a high risk of bias

**Supplementary Figure 8.** Funnel plot of the prevalence of (A) CF, (B) PRCF, and (C) RCF

**Supplementary Table 1.** PRISMA 2020 Checklist

| **Section and Topic** | **Item #** | **Checklist item** | **Location where item is reported** |
| --- | --- | --- | --- |
| **TITLE** | | |  |
| Title | 1 | Identify the report as a systematic review. | Title |
| **ABSTRACT** | | |  |
| Abstract | 2 | See the PRISMA 2020 for Abstracts checklist. | Abstract |
| **INTRODUCTION** | | |  |
| Rationale | 3 | Describe the rationale for the review in the context of existing knowledge. | Introduction/Paragraph 1-3 |
| Objectives | 4 | Provide an explicit statement of the objective(s) or question(s) the review addresses. | Introduction/Paragraph 4 |
| **METHODS** | | |  |
| Eligibility criteria | 5 | Specify the inclusion and exclusion criteria for the review and how studies were grouped for the syntheses. | Methods/Paragraph 3 |
| Information sources | 6 | Specify all databases, registers, websites, organisations, reference lists and other sources searched or consulted to identify studies. Specify the date when each source was last searched or consulted. | Methods/Paragraph 2 |
| Search strategy | 7 | Present the full search strategies for all databases, registers and websites, including any filters and limits used. | Methods/Paragraph 2, Supplementary |
| Selection process | 8 | Specify the methods used to decide whether a study met the inclusion criteria of the review, including how many reviewers screened each record and each report retrieved, whether they worked independently, and if applicable, details of automation tools used in the process. | Methods/Paragraph 4 |
| Data collection process | 9 | Specify the methods used to collect data from reports, including how many reviewers collected data from each report, whether they worked independently, any processes for obtaining or confirming data from study investigators, and if applicable, details of automation tools used in the process. | Methods/Paragraph 4, Supplementary |
| Data items | 10a | List and define all outcomes for which data were sought. Specify whether all results that were compatible with each outcome domain in each study were sought (e.g. for all measures, time points, analyses), and if not, the methods used to decide which results to collect. | Methods/Paragraph 5, Supplementary |
|  | 10b | List and define all other variables for which data were sought (e.g. participant and intervention characteristics, funding sources). Describe any assumptions made about any missing or unclear information. | Methods/Paragraph 5, Supplementary |
| Study risk of bias assessment | 11 | Specify the methods used to assess risk of bias in the included studies, including details of the tool(s) used, how many reviewers assessed each study and whether they worked independently, and if applicable, details of automation tools used in the process. | Methods/Paragraph 6 |
| Effect measures | 12 | Specify for each outcome the effect measure(s) (e.g. risk ratio, mean difference) used in the synthesis or presentation of results. | Methods/Paragraph 5 |
| Synthesis methods | 13a | Describe the processes used to decide which studies were eligible for each synthesis (e.g. tabulating the study intervention characteristics and comparing against the planned groups for each synthesis (item #5)). | Methods/Paragraph 4-5 |
|  | 13b | Describe any methods required to prepare the data for presentation or synthesis, such as handling of missing summary statistics, or data conversions. | Methods/Paragraph 7-8 |
|  | 13c | Describe any methods used to tabulate or visually display results of individual studies and syntheses. | Methods/Paragraph 7-8 |
|  | 13d | Describe any methods used to synthesize results and provide a rationale for the choice(s). If meta-analysis was performed, describe the model(s), method(s) to identify the presence and extent of statistical heterogeneity, and software package(s) used. | Methods/Paragraph 7-8, Supplementary |
|  | 13e | Describe any methods used to explore possible causes of heterogeneity among study results (e.g. subgroup analysis, meta-regression). | Methods/Paragraph 7-8, Supplementary |
|  | 13f | Describe any sensitivity analyses conducted to assess robustness of the synthesized results. | Methods/Paragraph 9, Supplementary |
| Reporting bias assessment | 14 | Describe any methods used to assess risk of bias due to missing results in a synthesis (arising from reporting biases). | Methods/Paragraph 7-9 |
| Certainty assessment | 15 | Describe any methods used to assess certainty (or confidence) in the body of evidence for an outcome. | Methods/Paragraph 10 |
| **RESULTS** | | |  |
| Study selection | 16a | Describe the results of the search and selection process, from the number of records identified in the search to the number of studies included in the review, ideally using a flow diagram. | Results/Paragraph 1 |
|  | 16b | Cite studies that might appear to meet the inclusion criteria, but which were excluded, and explain why they were excluded. | Results/Paragraph 1 |
| Study characteristics | 17 | Cite each included study and present its characteristics. | Results/Paragraph 2-3, Supplementary |
| Risk of bias in studies | 18 | Present assessments of risk of bias for each included study. | Results/Paragraph 4 |
| Results of individual studies | 19 | For all outcomes, present, for each study: (a) summary statistics for each group (where appropriate) and (b) an effect estimate and its precision (e.g. confidence/credible interval), ideally using structured tables or plots. | Results/Paragraph 5-11 |
| Results of syntheses | 20a | For each synthesis, briefly summarise the characteristics and risk of bias among contributing studies. | Results/Paragraph 5-11 |
|  | 20b | Present results of all statistical syntheses conducted. If meta-analysis was done, present for each the summary estimate and its precision (e.g. confidence/credible interval) and measures of statistical heterogeneity. If comparing groups, describe the direction of the effect. | Results/Paragraph 5-11 |
|  | 20c | Present results of all investigations of possible causes of heterogeneity among study results. | Results/Paragraph 5-11 |
|  | 20d | Present results of all sensitivity analyses conducted to assess the robustness of the synthesized results. | Results/Paragraph 12 |
| Reporting biases | 21 | Present assessments of risk of bias due to missing results (arising from reporting biases) for each synthesis assessed. | Results/Paragraph 13 |
| Certainty of evidence | 22 | Present assessments of certainty (or confidence) in the body of evidence for each outcome assessed. | Results/Paragraph 13 |
| **DISCUSSION** | | |  |
| Discussion | 23a | Provide a general interpretation of the results in the context of other evidence. | Discussion/Paragraph 1-7 |
|  | 23b | Discuss any limitations of the evidence included in the review. | Discussion/Paragraph 8 |
|  | 23c | Discuss any limitations of the review processes used. | Discussion/Paragraph 8 |
|  | 23d | Discuss implications of the results for practice, policy, and future research. | Discussion/Paragraph 9 |
| **OTHER INFORMATION** | | |  |
| Registration and protocol | 24a | Provide registration information for the review, including register name and registration number, or state that the review was not registered. | Methods/Paragraph 1 |
|  | 24b | Indicate where the review protocol can be accessed, or state that a protocol was not prepared. | Methods/Paragraph 1 |
|  | 24c | Describe and explain any amendments to information provided at registration or in the protocol. | Methods/Paragraph 1 |
| Support | 25 | Describe sources of financial or non-financial support for the review, and the role of the funders or sponsors in the review. | Funding |
| Competing interests | 26 | Declare any competing interests of review authors. | Declaration of interest |
| Availability of data, code and other materials | 27 | Report which of the following are publicly available and where they can be found: template data collection forms; data extracted from included studies; data used for all analyses; analytic code; any other materials used in the review. | Data sharing |

**Supplementary Table 2.** Search strategies

| **PubMed** |
| --- |
| #1 ((((((((cognitive frailty[MeSH Terms]) OR (reversible cognitive frailty[Title/Abstract])) OR (potentially reversible cognitive frailty[Title/Abstract])) OR (cognitive decline[Title/Abstract])) OR (cognitive frailty[Title/Abstract])) OR (cognitive impairment[Title/Abstract])) OR (cognitive dysfunction[Title/Abstract])) OR (cognitive frail*[Title/Abstract])) OR (subjective cognitive decline[Title/Abstract])  #2 ((((((frailty[MeSH Terms]) ) OR (frailty[Title/Abstract])) OR (frailty syndrome[Title/Abstract])) OR (frail*[Title/Abstract])) OR (pre-frail*[Title/Abstract])) OR (prefrail*[Title/Abstract])  #3 (((elder*[Title/Abstract]) OR (older*[Title/Abstract])) OR (senior*[Title/Abstract])) OR (geriatric*[Title/Abstract])  #4 #1 AND #2 AND #3 |
| **Web of Science** |
| #1 AB=(elder*) OR AB=(older*) OR AB=(senior*) OR AB=(geriatric*) OR TI=(elder*) OR TI=(older*) OR TI=(senior*) OR TI=(geriatric*)  #2 AB=(frailty) OR AB=(frailty syndrome) OR AB=(frail*) OR AB=( pre-frail*) OR AB=( prefrail*) OR TI=(frailty) OR TI=(frailty) OR TI=(frailty) OR TI=(frailty) OR TI=(frailty syndrome) OR TI=(frail*) OR TI=(pre-frail*) OR TI=(prefrail*)  #3 AB=(reversible cognitive frailty) OR AB=(potentially reversible cognitive frailty) OR AB=(cognitive frailty) OR AB=(cognitive decline) OR AB=(cognitive impairment) OR AB=(cognitive dysfunction) OR AB=(cognitive frail*) OR TI=(reversible cognitive frailty) OR TI=(potentially reversible cognitive frailty) OR TI=(cognitive frailty) OR TI=(cognitive decline) OR TI=(cognitive impairment) OR TI=(cognitive dysfunction) OR TI=(cognitive frail*) OR AB=(subjective cognitive decline) OR TI=(subjective cognitive decline)  #4 #1 and #2 and #3 |
| **Cochrane** |
| #1 reversible cognitive frailty or potentially reversible cognitive frailty or cognitive frailty or cognitive decline or cognitive impairment or cognitive dysfunction or cognitive frail* or subjective cognitive decline  #2 MeSH descriptor: [Frailty] explode all trees  #3 frailty or frailty syndrome or frail* or pre-frail* or prefrail*  #4 #2 or #3  #5 elder* or older* or senior* or geriatric*  #6 #1 and #4 and #5 |
| **Embase** |
| #1 ‘cognitive frailty’/exp OR ‘reversible cognitive frailty’:ta,ab,kw OR ‘potentially reversible cognitive frailty’:ta,ab,kw OR ‘cognitive frailty’:ta,ab,kw OR ‘cognitive decline’:ta,ab,kw OR ‘cognitive impairment’:ta,ab,kw OR ‘cognitive dysfunction’:ta,ab,kw OR ‘cognitive frail*’:ta,ab,kw OR ‘subjective cognitive decline’:ta,ab,kw  #2 ‘frailty’/exp OR ‘frailty’:ta,ab,kw OR ‘frailty syndrome’:ta,ab,kw OR ‘frail*’:ta,ab,kw OR ‘pre-frail*’:ta,ab,kw OR ‘prefrail*’:ta,ab,kw  #3 ‘elder*’:ta,ab,kw OR ‘older*’:ta,ab,kw OR ‘senior*’:ta,ab,kw OR ‘geriatric*’:ta,ab,kw  #4 #1 and #2 and #3 |
| **Wanfang** |
| (全部:(老年)) and (题名或关键词:(衰弱) or 摘要:(衰弱)) and (题名或关键词:(可逆性认知衰弱) or 题名或关键词:(认知衰弱) or 题名或关键词:(认知障碍) or题名或关键词:(主观认知下降) or 摘要:(可逆性认知衰弱) or 摘要:(认知衰弱) or 摘要:(认知障碍) or摘要:(主观认知下降)) |
| **CNKI** |
| (TKA ='衰弱') and (TKA='老年' OR TKA='老年人') and (TKA='可逆性认知衰弱' OR TKA='认知衰弱' OR TKA='认知障碍' OR TKA='主观认知下降') |

**Supplementary Table 3.** Characteristics of the included studies

| **Citation** | **Study period** | **Sample size** | **Design** | **Mean age** | **Region** | **Setting** | **Female prop.** | **Dementia** | **Physical frailty** | **Cognitive impairment** | **SCD** | **CF** | **PRCF** | **RCF** | **Age-specific prevalence** |
| --- | --- | --- | --- | --- | --- | --- | --- | --- | --- | --- | --- | --- | --- | --- | --- |
| 1. Aliberti et al. ^1^ | 2006, 2008 | 7338 | cohort | 74.4 | US | community | 0.549 | cognitive test | FFP | immediate and delayed recall, serial subtractions by 7, backward count task |  | 5% |  |  |  |
| 2. Alkhodary et al. ^2^ | 2019 | 730 | cross-sectional |  | Malaysia | community | 0.516 | non-cognitive test | FFP | CDR |  | 5% |  |  | **CF:** 65-70 (3%), 71-75 (4%), 76-80 (9%), 81-85 (5%) |
| 3. Avila-Funes et al. ^3^ | 1999-2000 | 6030 | cohort | 74.1 | France | community | 0.612 | non-cognitive test | FFP | MMSE, Isaacs Set Test |  | 2% | 7% |  |  |
| 4. Bai et al. ^4^ | 2023 | 496 | cross-sectional | 70.1 | China | community | 0.522 | combine | FS | MoCA |  |  | 19% |  | **PRCF:** 60-69 (16%), 70-79 (18%), ≥80 (33%) |
| 5. Bekić et al. ^5^ | 2018 | 263 | cross-sectional | 71.2 | Croatia | community | 0.650 | non-cognitive test | FFP | MMSE |  | 7% | 8% |  |  |
| 6. Beauchet et al ^6^ | 2003-2005 | 1259 | cohort |  | Canada | community | 0.523 | cognitive test | FFP | immediate and delayed recalls of the three words |  | 2% | 13% |  |  |
| 7. Chen et al ^7^ | 2011-2013 | 521 | cohort | 72.7 | Taiwan | community | 0.524 | cognitive test | FFP | MoCA |  | 1% |  |  |  |
| 8. Casas-Herrero et al. ^8^ |  | 43 | cross-sectional | 91.9 | Spain | nursing home | 0.674 | non-cognitive test | FFP | MMSE, subjective report |  | 30% |  |  |  |
| 9. Chen et al. ^9^ | 2018 | 4067 | cross-sectional | 78.88 | China | community | 0.455 | non-cognitive test | FI | MMSE |  | 1% |  |  |  |
| 10. Chen et al. ^10^ | 2021-2022 | 247 | cross-sectional | 73.26 | China | community | 0.571 | non-cognitive test | FFP | MMSE | self-reported memory complaints |  | 11% | 28% |  |
| 11. Chen et al. ^11^ | 2020-2021 | 526 | cross-sectional | 68 | China | community | 0.549 | non-cognitive test | FFP | MoCA, CDR | self-reported memory complaints |  | 19% | 17% |  |
| 12.Choi et al. ^12^ | 2017 | 9894 | cross-sectional |  | South Korea | community | 0.572 | non-cognitive test | FS | MMSE |  | 2% |  |  | **CF:** 65-74 (1%), ≥75 (3%) |
| 13. Cui et al. ^13^ | 2020-2021 | 475 | cross-sectional | 72.2 | China | community | 0.493 | non-cognitive test | FS | MoCA, CDR, subjective report |  | 9% |  |  | **CF:** 60-70 (4%), 71-80 (11%), >80 (22%) |
| 14. Daou et al. ^14^ | 2019-2020 | 112 | cross-sectional | 73.0 | Lebanon | community | 0.652 | cognitive test | FFP | RUDAS |  | 6% |  |  |  |
| 15. Das et al. ^15^ | 2018-2020 | 510 | cross-sectional | 71.43 | India | community | 0.549 | non-cognitive test | FFP | MMSE |  | 22% |  |  |  |
| 16. Delrieu et al. ^16^ | 2008 | 1,617 | cross-sectional | 75.37 | France | hospital | 0.647 | non-cognitive test | FFP | CDR |  |  | 22% |  |  |
| 17. Esteban-Cornejo et al. ^17^ | 2000-2001 | 3677 | cohort | 71.50 | Spain | community | 0.560 | non-cognitive test | FS | MMSE |  |  | 23% |  |  |
| 18. Gaspar et al. ^18^ | 2019 | 250 | cross-sectional | 71.04 | Portugal | community | 0.716 | non-cognitive test | FFP | MoCA | EMQ | 8% | 15% | 14% |  |
| 19. Ghanbarnia et al. ^19^ | 2016-2017 | 1775 | cross-sectional | 69.72 | Iran | community | 0.471 | combine | FS | MMSE |  | 12% | 23% |  | **CF:** 60-64 (4%), 65-69 (8%), 70-74 (17%), 75-79 (20%), 80-84 (22%), 85-99 (27%) |
| 20. Gifford et al. ^20^ | 2012-2014 | 306 | cross-sectional | 73.0 | US | community | 0.422 | non-cognitive test | FFP | MoCA |  |  | 17% |  |  |
| 21. Hao et al. ^21^ | 2005 | 705 | cohort | 93.6 | China | community | 0.674 | non-cognitive test | FI | MMSE |  | 50% |  |  | **CF:** ≥90 (50%) |
| 22. Hao et al. ^22^ | 2022-2023 | 991 | cross-sectional |  | China | community | 0.535 | non-cognitive test | FS | CDR |  |  | 29% |  | **PRCF:** 60-69 (4%), 70-79 (33%), ≥80 (74%) |
| 23. Hou et al. ^23^ | 2020 | 527 | cross-sectional |  | China | community | 0.505 | non-cognitive test | FS | MMSE |  |  | 20% |  | **PRCF:** 60-69 (17%), ≥70 (23%) |
| 24. Huang et al. ^24^ | 2021 | 1279 | cross-sectional | 72.68 | China | community | 0.589 | non-cognitive test | FFP | MMSE, subjective report | simplified SCD questionnaire |  |  | 38% | **RCF:** 60-69 (38%), 70-79 (39%), ≥80 (32%) |
| 25. Huang et al. ^25^ | 2014–2016 | 1115 | cross-sectional |  | Taiwan | community | 0.528 | non-cognitive test | FS | MMSE |  | 4% | 24% |  |  |
| 26. Inoue et al. ^26^ | 2017-2019 | 432 | cross-sectional | 75.9 | Japan | hospital | 0.690 | combine | FFP | MoCA |  | 21% |  |  |  |
| 27. Jang et al. ^27^ | 2020 | 386 | cross-sectional | 76.31 | South Korea | community | 0.671 | cognitive test | FFP | MMSE |  | 8% | 17% |  |  |
| 28. Jiang et al. ^28^ | 2020-2022 | 442 | cross-sectional | 71.1 | China | hospital | 0.532 | non-cognitive test | FFP | MMSE |  | 5% | 19% |  |  |
| 29. Jing et al. ^29^ | 2019 | 3242 | cross-sectional | 70.1 | China | community | 0.535 | non-cognitive test | FFP | MMSE |  | 7% |  |  |  |
| 30. Kamasaki et al. ^30^ | 2019-2021 | 121 | cross-sectional | 77.0 | Japan | community | 0.744 | cognitive test | slow walking speed or/and muscle weakness | MMSE |  | 12% |  |  |  |
| 31. Katayama et al. ^31^ | 2015-2018 | 8003 | cross-sectional | 72.5 | Japan | community | 0.561 | combine | slow walking speed or/and muscle weakness | NCGG-FAT |  | 11% |  |  |  |
| 32. Kawamura et al. ^32^ | 2021 | 464 | cross-sectional | 76.3 | Japan | community | 0.603 | non-cognitive test | KCL | SDC |  | 3% | 4% |  |  |
| 33. Kitro et al. ^33^ | 2022-2023 | 984 | cross-sectional | 69.8 | Thailand | community | 0.622 | non-cognitive test | FS | MMSE |  | 5% |  |  |  |
| 34. Ko et al. ^34^ | 2020 | 9827 | cross-sectional | 73.41 | South Korea | community | 0.599 | non-cognitive test | FS | MMSE |  | 3% |  |  | **CF:** 65-74 (1%), 75-84 (4%), ≥85 (11%) |
| 35. Kwan et al. ^35^ | 2017-2018 | 185 | cross-sectional | 86.2 | Hong Kong | community | 0.714 | combine | FS | CDR |  |  | 36% |  |  |
| 36. Lee et al. ^36^ | 2016-2017 | 2028 | cross-sectional | 75.9 | South Korea | community | 0.499 | combine | FFP | MMSE |  | 3% |  |  |  |
| 37. Li et al. ^37^ | 2013 | 2693 | cross-sectional |  | Taiwan | community | 0.524 | non-cognitive test | FS | MMSE |  |  | 12% |  | **PRCF:** 65-74 (7%), ≥75 (19%) |
| 38. Li et al. ^38^ | 2020 | 1458 | cross-sectional | 76.42 | China | community | 0.508 | non-cognitive test | FFP | MMSE |  | 11% |  |  | **CF:** 65-75 (9%), ≥75 (14%) |
| 39.Li et al. ^39^ | 2023 | 108 | cross-sectional | 74.5 | China | hospital | 0.556 | combine | FS | MMSE |  |  | 36% |  | **PRCF:** 60-69 (24%), 70-79 (35%), ≥80 (56%) |
| 40.Limpawattana et al. ^40^ | 2019-2022 | 198 | cross-sectional |  | Thailand | hospital | 0.626 | non-cognitive test | FI | MoCA |  | 21% |  |  |  |
| 41. Liu et al. ^41^ | 2021 | 1206 | cross-sectional | 77.32 | China | nursing home | 0.505 | non-cognitive test | FS | MMSE |  |  | 17% |  | **PRCF:** 60-69 (16%), 70-79 (17%), 80-89 (17%), ≥90 (28%) |
| 42. Liu et al. ^42^ | 2019 | 1006 | cross-sectional | 74.8 | China | hospital | 0.542 | non-cognitive test | FS | MoCA, CDR, subjective report |  | 13% |  |  | **CF:** 60-74 (7%), 75-89 (18%), ≥90 (38%) |
| 43. Lu et al. ^43^ | 2019 | 3558 | cross-sectional | 69.9 | China | community | 0.489 | non-cognitive test | FS | MMSE |  | 4% | 10% |  |  |
| 44. Ma et al. ^44^ | 2018-2019 | 1412 | cross-sectional | 72.9 | China | hospital | 0.538 | non-cognitive test | FS | Mini-Cog |  | 7% | 34% |  |  |
| 45. Ma et al. ^45^ | 2011-2012 | 3202 | cross-sectional | 70.14 | China | community | 0.561 | non-cognitive test | FFP | MMSE |  | 2% | 5% |  |  |
| 46. Ma et al. ^46^ | 2014 | 1607 | cross-sectional | 75.2 | China | community | 0.523 | cognitive test | FFP | HDS-R |  | 3% | 11% |  | **CF:** 70-74 (2%), 75-79 (3%), 80-84 (5%) |
| 47. Rivan et al. ^47^ | 2012-2013 | 815 | cross-sectional | 68.9 | Malaysia | community | 0.544 | cognitive test | FFP | Digit Span, Verbal Learning Test, MMSE |  | 2% | 40% |  |  |
| 48. Rivan et al. ^48^ | 2016-2017 | 1318 | cross-sectional | 72.1 | Malaysia | community | 0.551 | cognitive test | FFP | Digit Span, Verbal Learning Test, MMSE |  |  | 37% |  |  |
| 49. Maruta et al. ^49^ | 2018-2019 | 882 | cross-sectional | 74.4 | Japan | community | 0.621 | non-cognitive test | KCL | NCGG-FAT |  | 3% | 17% |  |  |
| 50.Merchant et al. ^50^ | 2017-2019 | 509 | cross-sectional | 73.0 | Singapore | community | 0.802 | non-cognitive test | FS | MMSE | self-reported memory complaints | 1% | 9% | 29% |  |
| 51.Montero-Odasso et al. ^51^ | 2007-2015 | 252 | cohort | 76.7 | Canada | community | 0.627 | non-cognitive test | FFP | MoCA |  | 11% | 37% |  |  |
| 52. Navarro-Pardo et al. ^52^ | 2018-2019 | 285 | cross-sectional |  | Spain | community | 0.537 | non-cognitive test | FFP | MoCA |  | 3% | 22% |  | **PRCF:** 60-64 (13%), 65-69 (13%), 70-74 (27%), 75-79 (18%), ≥80 (44%) |
| 53. Pan et al. ^53^ | 2018 | 1190 | cross-sectional | 72.5 | China | community | 0.628 | non-cognitive test | FFP | MoCA, CDR |  |  | 10% |  | **PRCF:** 65-74 (2%), 75-84 (14%), 85-96 (23%) |
| 54. Razjouyan et al. ^54^ |  | 163 | cross-sectional | 75.0 | US | community | 0.790 | cognitive test | FFP | MMSE |  |  | 18% |  |  |
| 55. Ruan et al. ^55^ | 2018-2019 | 5076 | cross-sectional | 71.36 | China | community | 0.535 | cognitive test | FS | RCS | simplified SCD questionnaire |  | 6% | 20% | **PRCF:** 60-69 (2%), 70-79 (5%), ≥80 (21%)  **RCF:** 60-69 (16%), 70-79 (20%), ≥80 (31%) |
| 56. Ruan et al. ^56^ | 2018-2019 | 335 | cross-sectional |  | China | community | 0.448 | non-cognitive test | FFP | CDR, MMSE, subjective report | SCD questionnaire MyCog scores |  | 13% | 13% |  |
| 57. Sardone et al. ^57^ | 2014 | 1929 | cross-sectional | 73.6 | Italy | community | 0.495 | non-cognitive test | FFP | MMSE |  | 5% |  |  |  |
| 58. Seesen et al. ^58^ | 2021 | 373 | cross-sectional | 70.5 | Thailand | community | 0.584 | combine | FFP | MoCA |  |  | 36% |  |  |
| 59. Shen et al. ^59^ | 2022 | 506 | cross-sectional | 69 | China | hospital | 0.453 | non-cognitive test | TFI | MMSE, CDR |  | 29% |  |  | **CF:** 60-69 (9%), 70-79 (39%), ≥80 (76%) |
| 60. Shi et al. ^60^ | 2018-2021 | 158 | cross-sectional |  | China | hospital | 0.247 | non-cognitive test | FFP | MMSE |  |  | 22% |  |  |
| 61. Shimada et al. ^61^ | 2011-2013 | 8864 | cross-sectional | 73.4 | Japan | community | 0.520 | combine | FFP | NCGG-FAT |  | 1% |  |  |  |
| 62. Shirooka et al. ^62^ |  | 483 | cross-sectional | 73.3 | Japan | community | 0.683 | non-cognitive test | FFP | MMSE |  | 6% |  |  |  |
| 63.Sleight et al. ^63^ | 2011-2012 | 457 | cross-sectional | 76.50 | US | community | 0.571 | non-cognitive test | FFP | RBANS |  | 3% |  |  |  |
| 64. Solfrizzi et al. ^64^ | 1992-1993 | 2373 | cohort |  | Italy | community | 0.445 | combine | FFP | MMSE, Babcock Story Recall Test |  | 1% |  |  |  |
| 65. Song et al. ^65^ | 2019 | 1652 | cross-sectional | 71.4 | China | community | 0.581 | non-cognitive test | FFP | MMSE |  | 3% |  |  | **CF:** 60-69 (2%), 70-79 (4%), ≥80 (7%) |
| 66. Sugimoto et al. ^66^ | 2010-2017 | 333 | cross-sectional | 74.7 | Japan | hospital | 0.619 | non-cognitive test | FFP | MMSE |  |  | 59% |  |  |
| 67. Suprawesta et al. ^67^ | 2017-2019 | 832 | cross-sectional | 70.9 | Taiwan | hospital | 0.657 | non-cognitive test | FFP | CDR | self-reported memory complaints |  | 14% | 48% |  |
| 68. Safien et al. ^68^ | 2021-2022 | 755 | cross-sectional |  | Malaysia | community | 0.592 | non-cognitive test | FFP | CDR |  |  | 39% |  |  |
| 69. Tang et al. ^69^ | 2014 | 653 | cross-sectional | 72 | China | community | 0.613 | cognitive test | FS | MMSE |  | 2% |  |  |  |
| 70. Tseng et al. 1 ^70^ |  | 724 | cross-sectional | 73.1 | Taiwan | community | 0.468 | cognitive test | slow walking speed or/and muscle weakness | Verbal Learning Test, Boston Naming Test, Verbal Fluency Test, Taylor Complex Figure Test, Digit Backward Test, Clock Drawing Test |  | 21% |  |  | **CF:** 65-74 (15%), ≥75 (33%) |
| 71. Tseng et al. 2 ^70^ |  | 547 | cross-sectional |  | Taiwan | community |  | cognitive test | slow walking speed or/and muscle weakness | MoCA |  | 8% |  |  |  |
| 72. Wang et al. ^71^ | 2018 | 268 | cross-sectional | 80.59 | China | nursing home | 0.519 | non-cognitive test | FFP | MMSE, CDR |  | 18% |  |  | **CF:** 60-74 (2%), 75-96 (25%) |
| 73. Wang et al. ^72^ | 2023 | 154 | cross-sectional | 67 | China | hospital | 0.403 | non-cognitive test | FS | MMSE |  |  | 53% |  |  |
| 74. Wang et al. ^73^ | 2019-2020 | 486 | cross-sectional | 77.99 | China | hospital | 0.342 | non-cognitive test | FS | MMSE |  |  | 37% |  |  |
| 75. Wu et al. ^74^ | 2020-2021 | 643 | cross-sectional | 71.5 | China | community | 0.585 | non-cognitive test | FFP | MMSE | self-reported memory complaints | 3% | 18% | 15% | **CF:** 60-70 (1%), 71-80 (3%), >80 (12%)  **PRCF:** 60-70 (11%), 71-80 (19%), >80 (43%)  **RCF:** 60-70 (16%), 71-80 (16%), >80 (9%) |
| 76. Xie et al. ^75^ | 2018 | 1585 | cross-sectional | 81.4 | China | community | 0.582 | non-cognitive test | FFP | MMSE |  | 7% |  |  | **CF:** 75-80 (3%), 81-85 (7%), >85 (16%) |
| 77. Yang et al. ^76^ | 2014-2015 | 329 | cross-sectional | 79.6 | China | hospital | 0.416 | cognitive test | CFS | MMSE |  | 7% |  |  |  |
| 78. Yao et al. ^77^ | 2020-2021 | 955 | cross-sectional | 68.1 | China | community | 0.555 | non-cognitive test | FS | ﻿AD-8 |  |  | 9% |  | **PRCF:** 60-69 (7%), 70-79 (10%), ≥80 (16%) |
| 79. Yassuda et al ^78^ | 2009 | 384 | cross-sectional | 72.3 | Brazil | community | 0.602 | non-cognitive test | FFP | MMSE |  | 3% | 13% |  |  |
| 80. Yasuoka et al. ^79^ | 2015-2020 | 550 | cohort |  | Japan | hospital | 0.313 | cognitive test | FS | MMSE |  | 26% | 37% |  |  |
| 81. Yi et al. ^80^ | 2007-2008 | 39148 | cohort | 66 | South Korea | community | 0.533 | non-cognitive test | Timed Up and Go test | KDSQ-P |  | 9% |  |  |  |
| 82. Yoon et al. ^81^ | 2015 | 104 | cross-sectional | 73.5 | South Korea | community | 0.769 | cognitive test | FFP | MMSE |  | 16% | 52% |  |  |
| 83. Yue et al. ^82^ | 2019 | 445 | cross-sectional | 76.96 | China | hospital | 0.333 | non-cognitive test | FS | MMSE |  |  | 34% |  |  |
| 84. Zeng et al. ^83^ | 2014-2018 | 311 | cohort |  | China | hospital | 0.415 | non-cognitive test | CFS | MMSE |  | 16% |  |  |  |
| 85. Zhang et al. ^84^ | 2020 | 2638 | cross-sectional |  | China | community | 0.638 | non-cognitive test | FFP | MMSE |  | 8% |  |  |  |
| 86. Zhang et al. ^85^ | 2018-2019 | 9194 | cross-sectional | 72.41 | China | hospital | 0.419 | non-cognitive test | FS | Mini-Cog |  | 5% |  |  |  |
| 87. Zhang et al. ^86^ | 2022 | 452 | cross-sectional | 80.9 | China | nursing home | 0.655 | non-cognitive test | FFP | MoCA | self-reported memory complaints | 19% | 50% | 15% |  |
| 88. Zhao et al. ^87^ | 2018 | 4093 | cross-sectional | 67.8 | China | community | 0.583 | non-cognitive test | FFP | SPMSQ |  | 3% | 12% |  | **PRCF:** 60-69 (8%), 70-79 (17%), ≥80 (28%) |
| 89. Zhou et al. ^88^ | 2019 | 303 | cross-sectional | 81.9 | China | nursing home | 0.548 | non-cognitive test | FFP | MoCA, CDR |  | 27% |  |  | **CF:** 60-69 (17%), 70-79 (17%), 80-89 (29%), ≥90 (46%) |
| 90. Zoghbi et al. ^89^ | 2012 | 111 | cross-sectional | 76.29 | Lebanon | nursing home | 0.505 | cognitive test | SOF | MMSE |  | 23% |  |  |  |

Abbreviations: CF: cognitive frailty; PRCF: potentially reversible cognitive frailty; RCF: reversible cognitive frailty; FFP: Fried Frailty Phenotype; FS: Frailty Scale; FI: Frailty Index; CFS: Clinical Frailty Scale; KCL: Kihon checklist; SOF: Study of Osteoporotic Fractures index; TFI: Tilburg Frailty Index; MMSE: Mini Mental State Examination; MoCA: Montreal Cognitive Assessment; CDR: Clinical Dementia Rating Scale; NCGG-FAT: National Centre for Geriatrics and the Gerontology-Functional Assessment Tool; RUDAS: Rowland Universal Dementia Assessment Scale; AD-8: Ascertain Dementia-8; HDS-R: Revised Hasegawa dementia scale; KDSQP: Korean Dementia Screening Questionnaires-Pre-screening; RBANS: Repeatable Battery for the Assessment of Neuropsychological Status; RCS: Rapid Cognitive Screen; SDC: self-administered dementia checklist; SPMSQ: Short Portable Mental Status Questionnaire; EMQ: Everyday Memory Questionnaire

**Supplementary Table 4.** Assessment tools used in the included studies

| **Assessment tools** | **Included studies** |
| --- | --- |
| Fried Frailty Phenotype ^90^ | ^1-3, 5-8, 10, 11, 13-16, 18, 20, 24, 26-29, 36, 38, 45, 46, 48, 51-54, 56-58, 60-68, 71, 74, 75, 78, 81, 84, 86-88^ |
| FRAIL scale ^91^ | ^4, 12, 17, 19, 22, 23, 25, 33-35, 37, 39, 41-44, 50, 55, 69, 72, 73, 77, 79, 82, 85^ |
| Slow walking speed and/or muscle weakness ^92^ | ^30, 31, 70^ |
| Frailty Index ^93^ | ^9, 21, 40^ |
| Clinical Frailty Scale ^94^ | ^76, 83^ |
| Kihon Checklist ^95^ | ^32, 49^ |
| Study of Osteoporotic Fractures Index ^96^ | ^89^ |
| Timed Up and Go test ^97^ | ^80^ |
| Tilburg Frailty Index ^#^ ^98^ | ^59^ |
| Mini Mental State Examination ^99^ | ^5, 9, 10, 12, 15, 17, 19, 21, 23, 25, 27-30, 33, 34, 36-39, 41, 43, 45, 50, 54, 57, 60, 62, 65, 66, 69, 72-76, 78, 79, 81-84, 89^ |
| Montreal Cognitive Assessment ^100^ | ^4, 7, 18, 20, 26, 40, 51, 52, 58, 70, 86^ |
| Clinical Dementia Rating Scale ^101^ | ^2, 16, 22, 35, 67, 68^ |
| National Center for Geriatrics and the Gerontology-Functional Assessment Tool ^102^ | ^31, 49, 61^ |
| Mini-Cog ^103^ | ^44, 85^ |
| Rowland Universal Dementia Assessment Scale ^104^ | ^14^ |
| Ascertain Dementia-8 ^105^ | ^77^ |
| Hasegawa Dementia Scale ^106^ | ^46^ |
| Korean Dementia Screening Questionnaires-Prescreening ^107^ | ^80^ |
| Repeatable Battery for the Assessment of Neuropsychological Status ^108^ | ^63^ |
| Rapid Cognitive Screen ^109^ | ^55^ |
| Self-administered dementia checklist ^110^ | ^32^ |
| Short Portable Mental Status Questionnaire ^111^ | ^87^ |
| Immediate and delayed recalls ^112^ | ^6^ |
| Self-reported memory complaints ^113^ | ^10, 11, 50, 67, 74, 86^ |
| Simplified SCD questionnaire ^114^ | ^24, 55^ |
| Everyday Memory Questionnaire ^115^ | ^18^ |
| SCD questionnaire MyCog scores ^116^ | ^56^ |

Note: ^#^ The Tilburg Frailty Index comprises physical, psychological, and social domains. The psychological domain includes self-reported memory problems alongside measures of emotional status and coping ability. While the memory item may overlap conceptually with cognitive assessments, it reflects subjective perception rather than objective cognitive performance.

**Supplementary Figure 1.** Definitions of cognitive frailty by IANA/IAGG, as well as those for potentially reversible cognitive frailty and reversible cognitive frailty by Ruan et al

Abbreviations: IANA: International Academy of Nutrition and Aging; IAGG: International Association of Gerontology


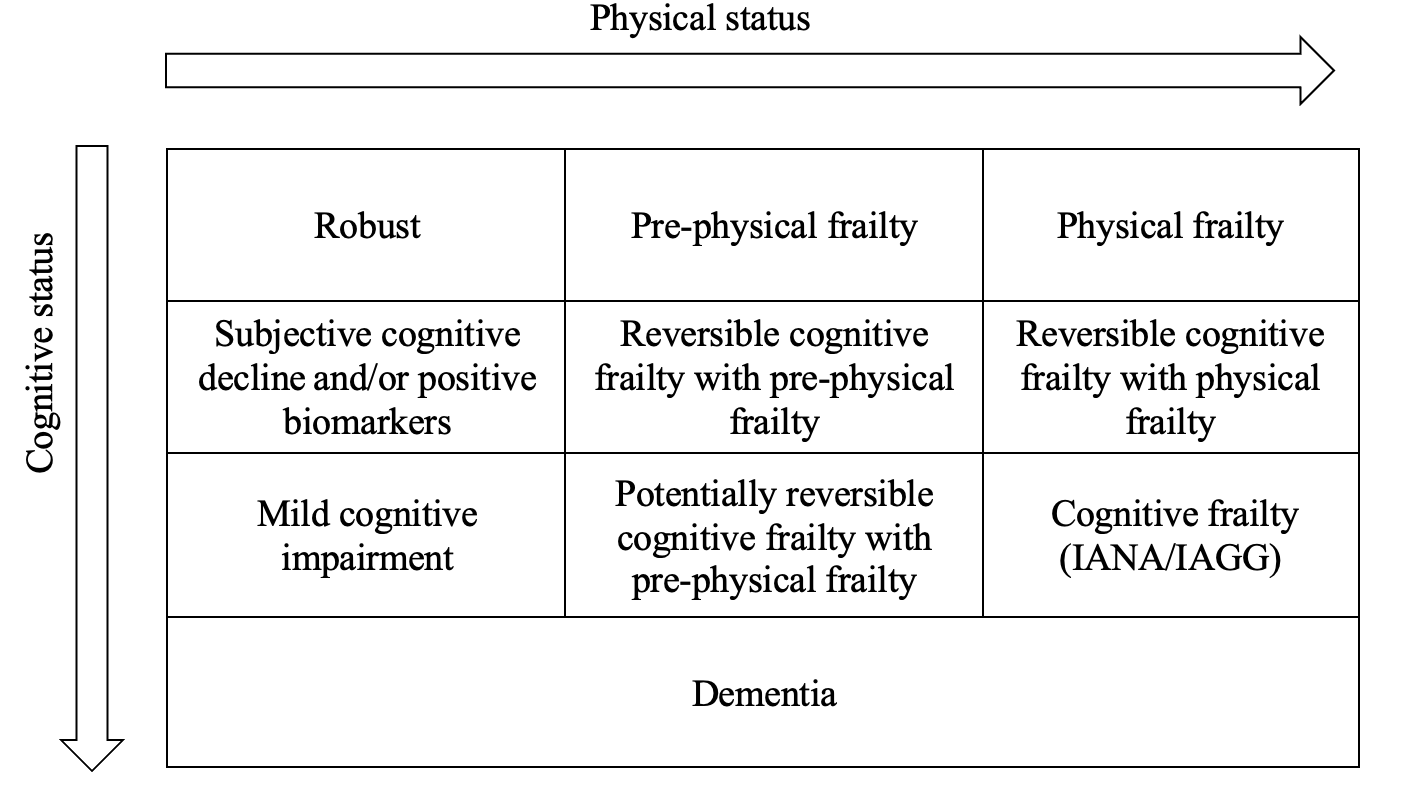


**Supplementary Figure 2.** Risk of bias assessment for the included studies for (A) CF, (B) PRCF, and (C) RCF

Abbreviations: CF: cognitive frailty; PRCF: potentially reversible cognitive frailty; RCF: reversible cognitive frailty

**(A) CF**


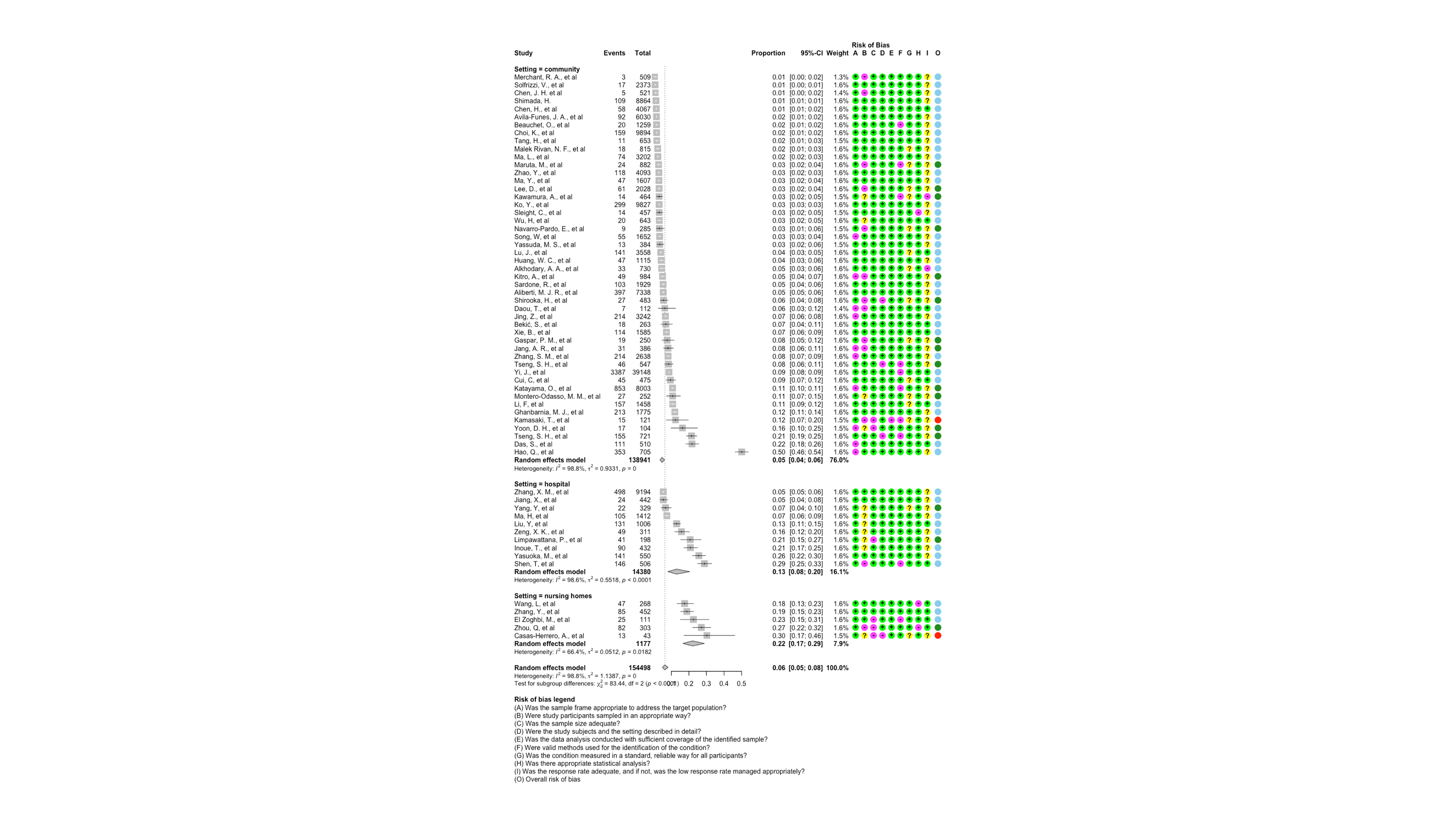


**(B) PRCF**


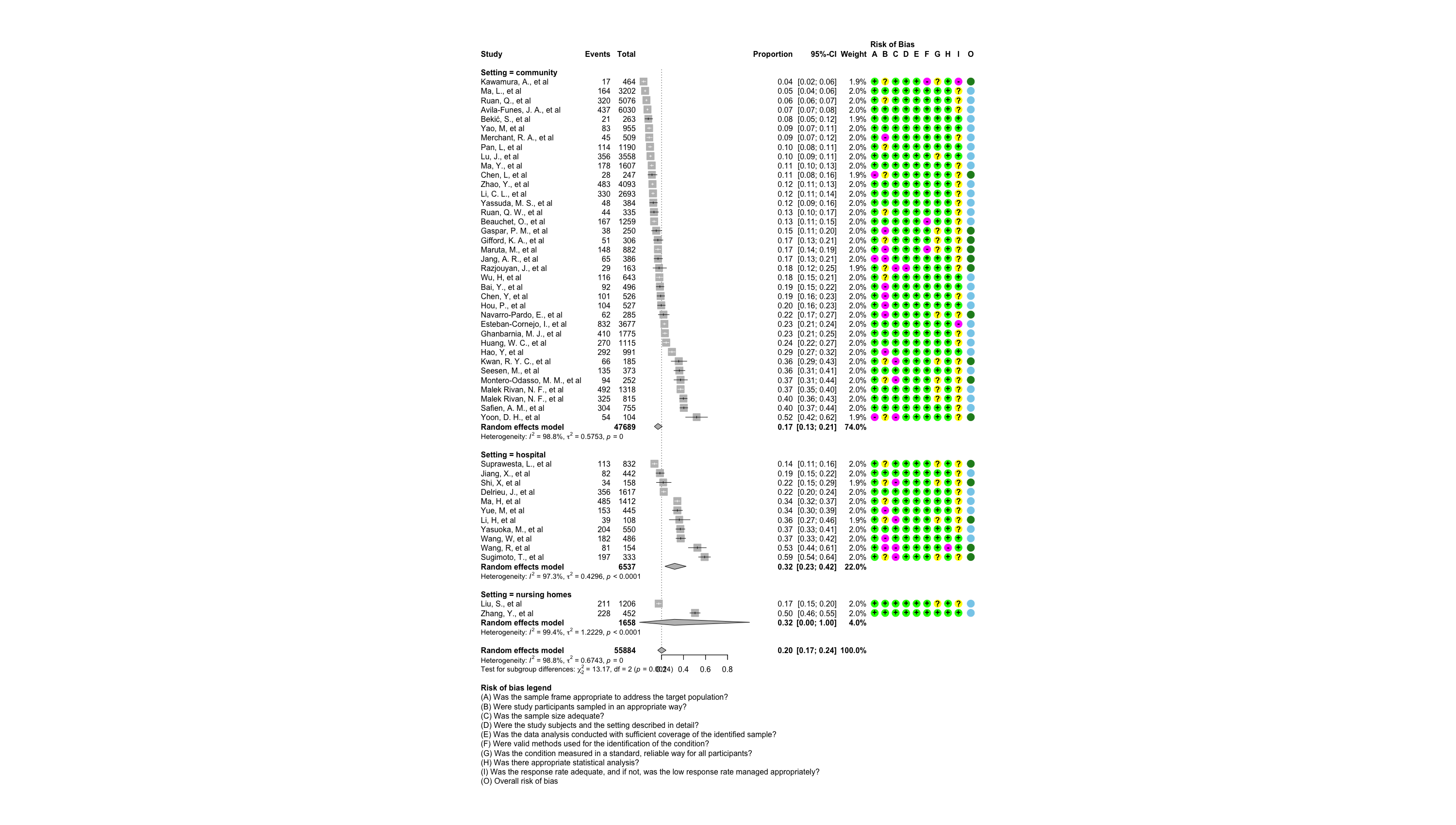


**(C) RCF**


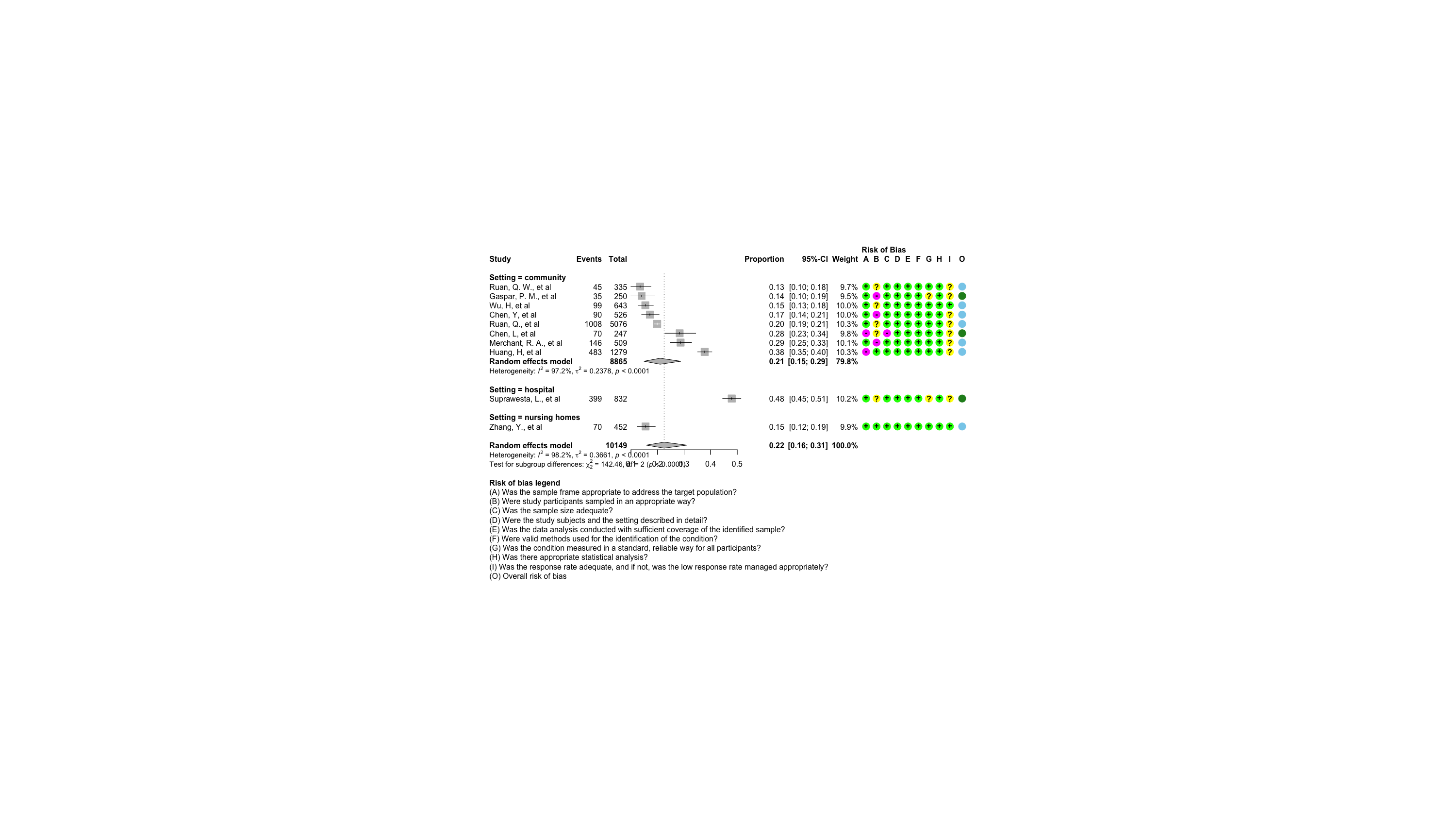


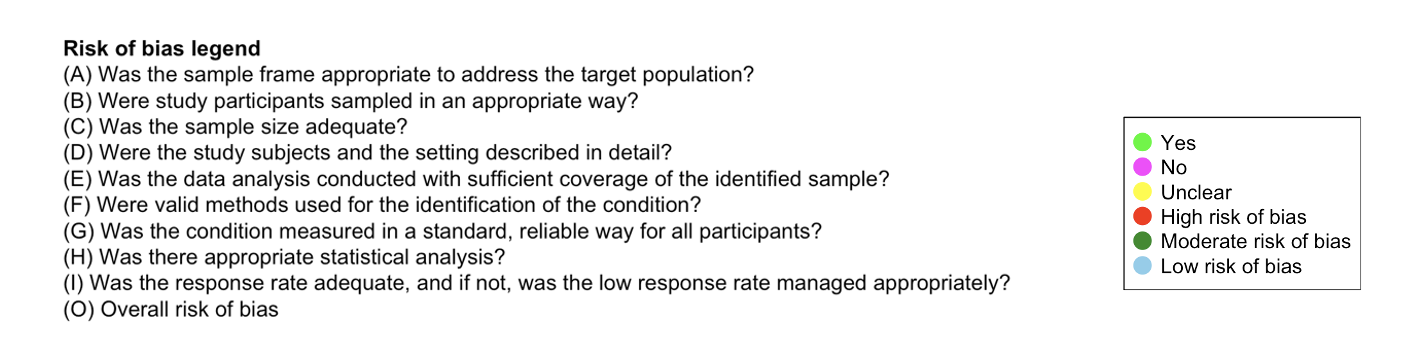


**Supplementary Figure 3.** Subgroup analyses for prevalence of (A) cognitive frailty, (B) potentially reversible cognitive frailty, and (C) reversible cognitive frailty (full versions of Figures 2 and 3 in the article).


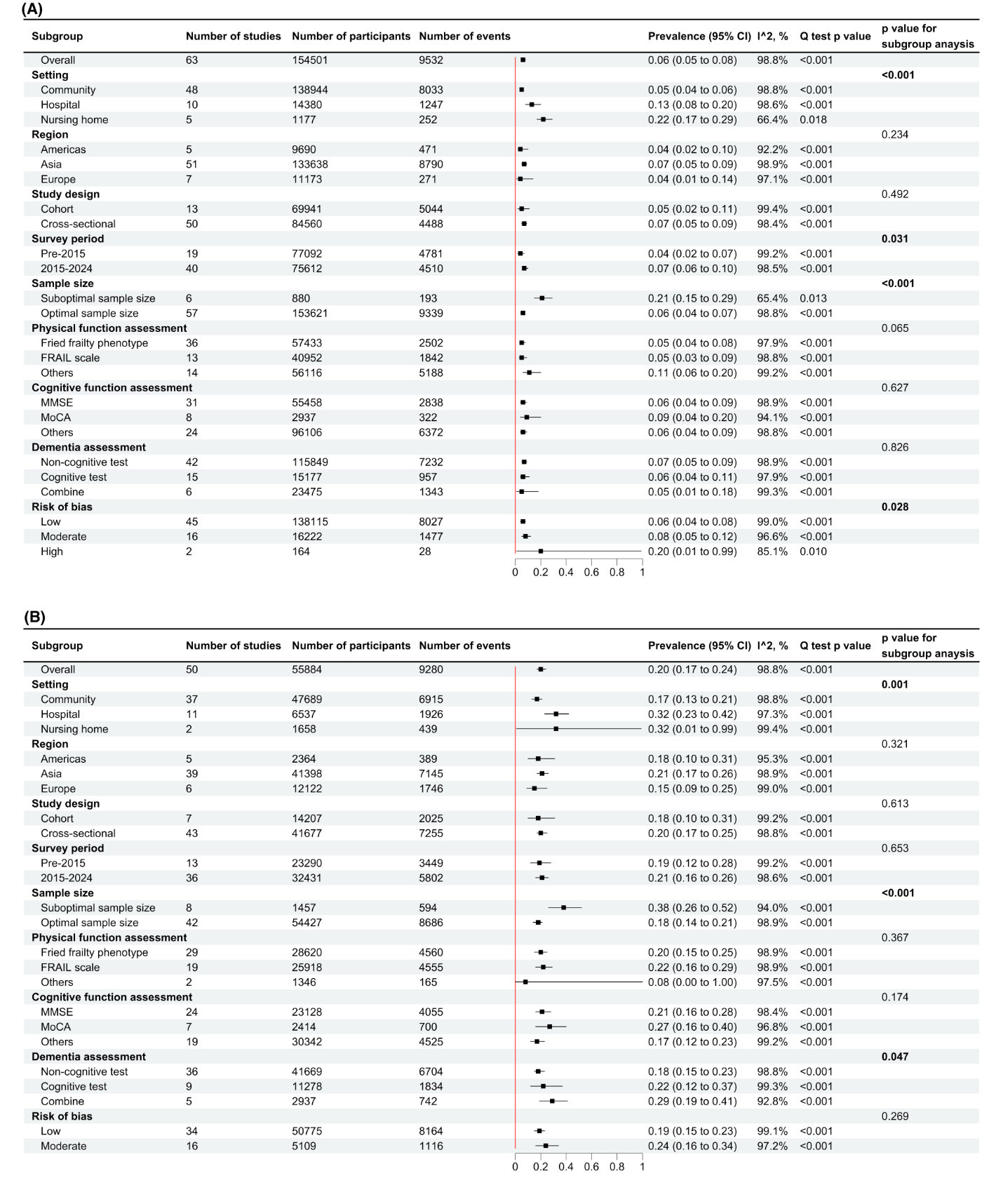


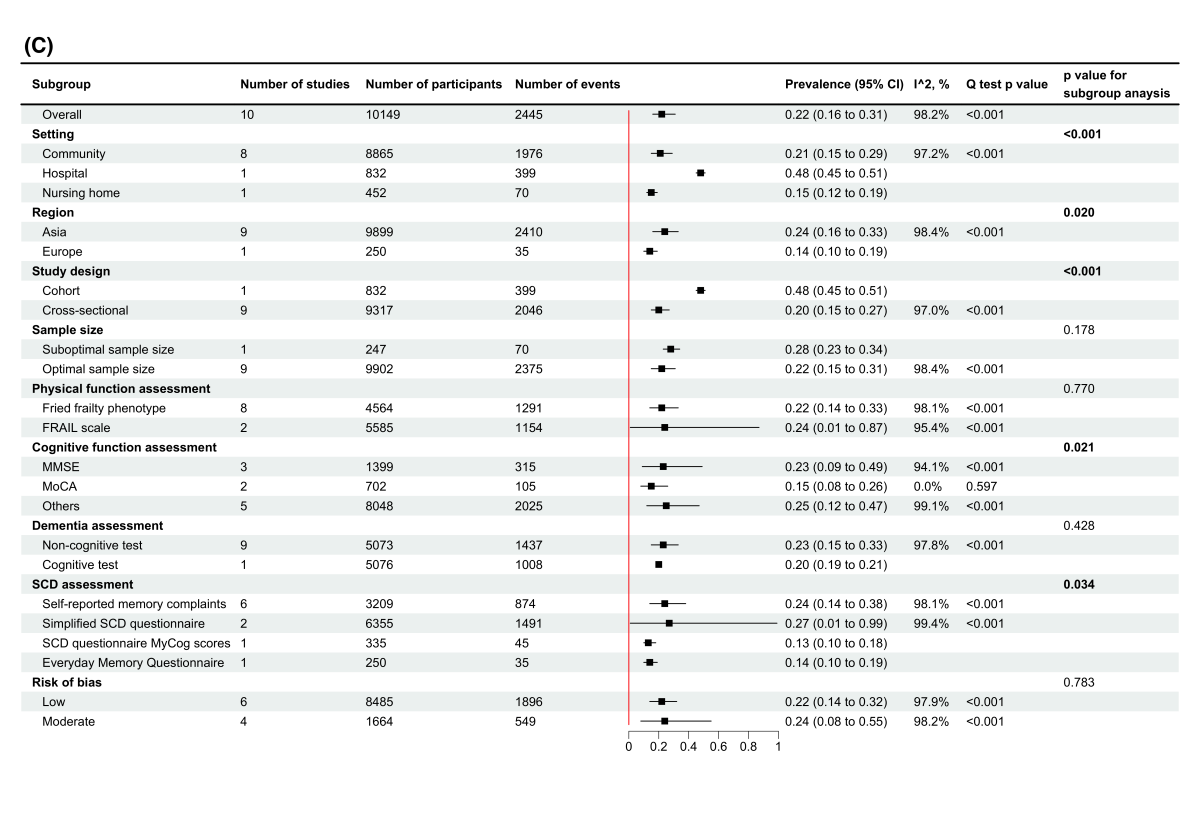


**Supplementary Figure 4.** Sensitivity analysis using the Leave-one-out approach for (A) CF, (B) PRCF, and (C) RCF

Abbreviations: CF: cognitive frailty; PRCF: potentially reversible cognitive frailty; RCF: reversible cognitive frailty

**(A) CF**


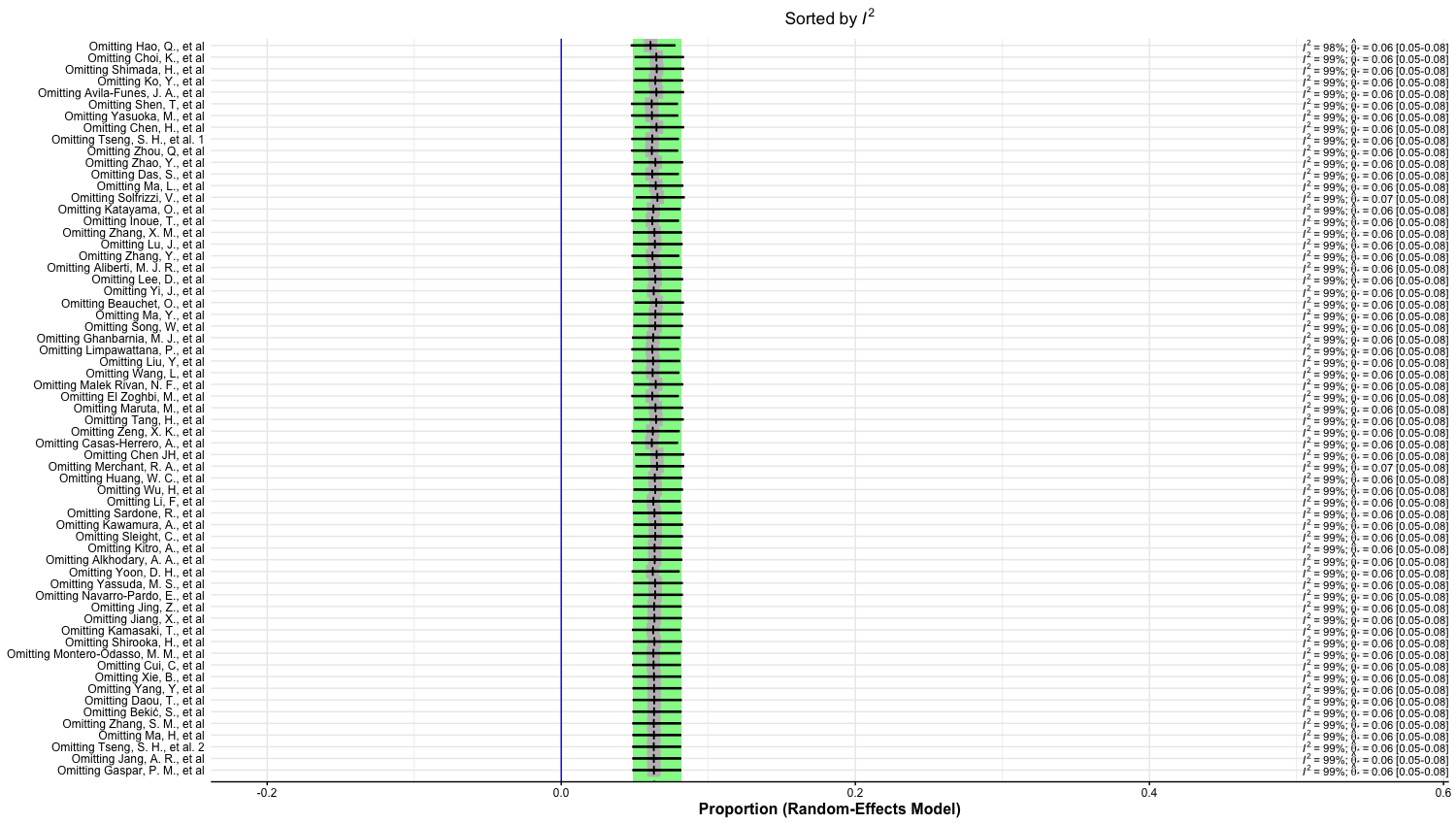


**(B) PRCF**


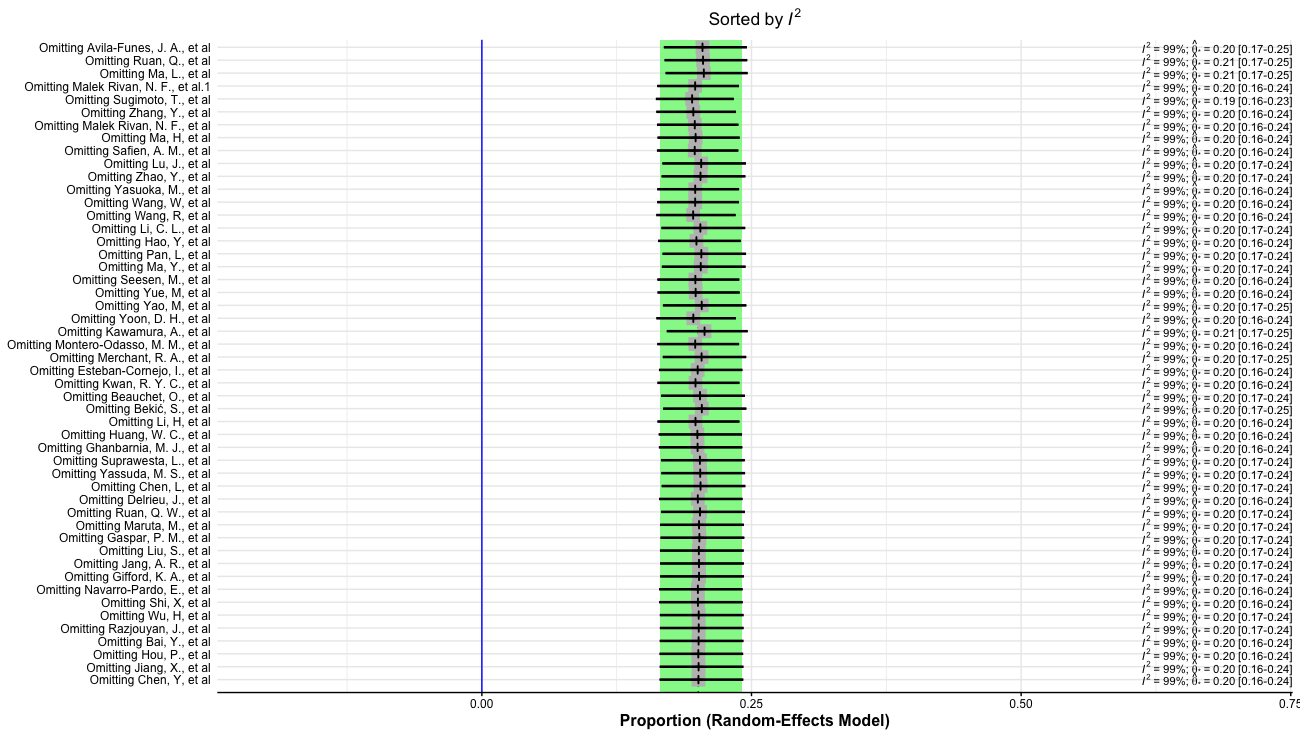


**(C) RCF**


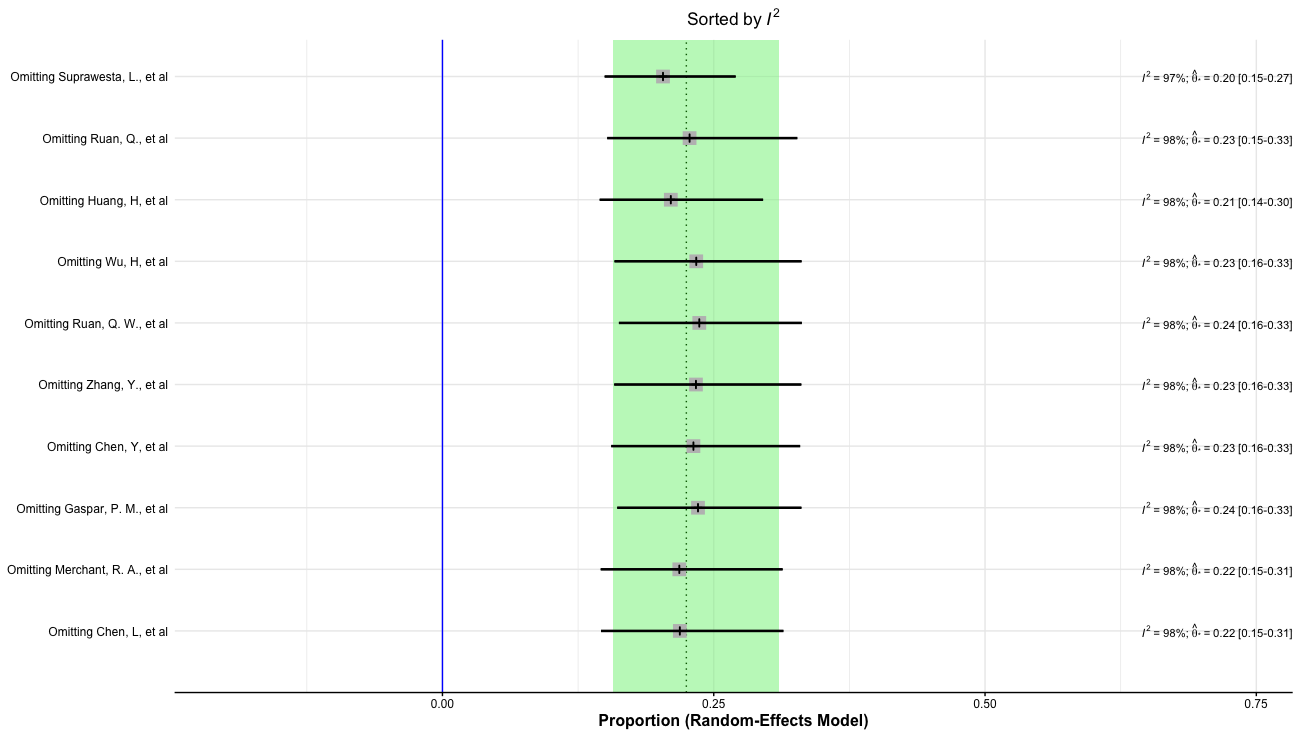


**Supplementary Figure 5.** Baujat plots of (A) CF, (B) PRCF, and (C) RCF

Abbreviations: CF: cognitive frailty; PRCF: potentially reversible cognitive frailty; RCF: reversible cognitive frailty

**(A) CF**

**
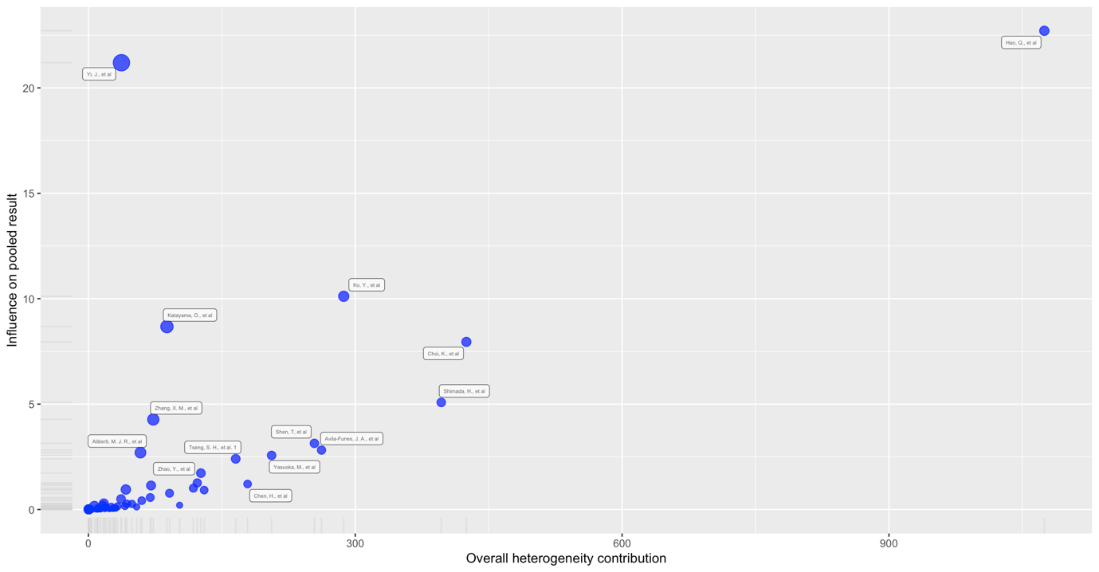
**

**(B) PRCF**

**
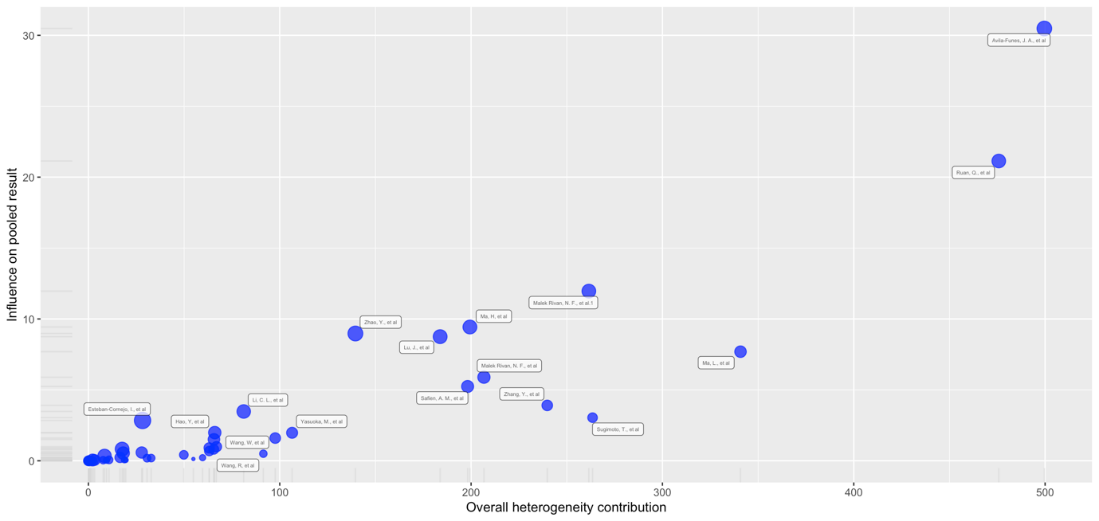
**

**(C) RCF**

**
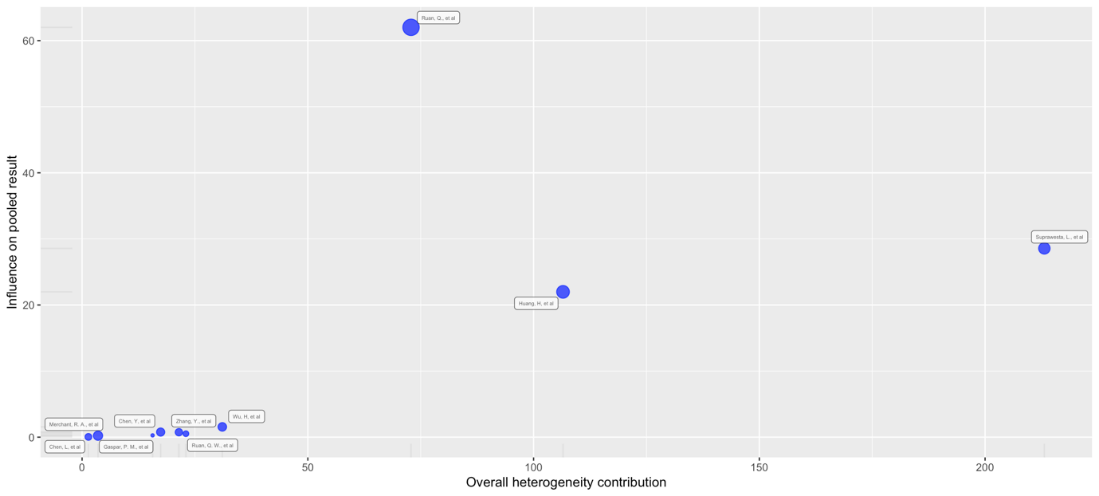
**

**Supplementary Figure 6.** Prevalence of (A) CF, (B) PRCF, and (C) RCF after excluding studies with high heterogeneity contribution

Abbreviations: CF: cognitive frailty; PRCF: potentially reversible cognitive frailty; RCF: reversible cognitive frailty

**(A) CF**


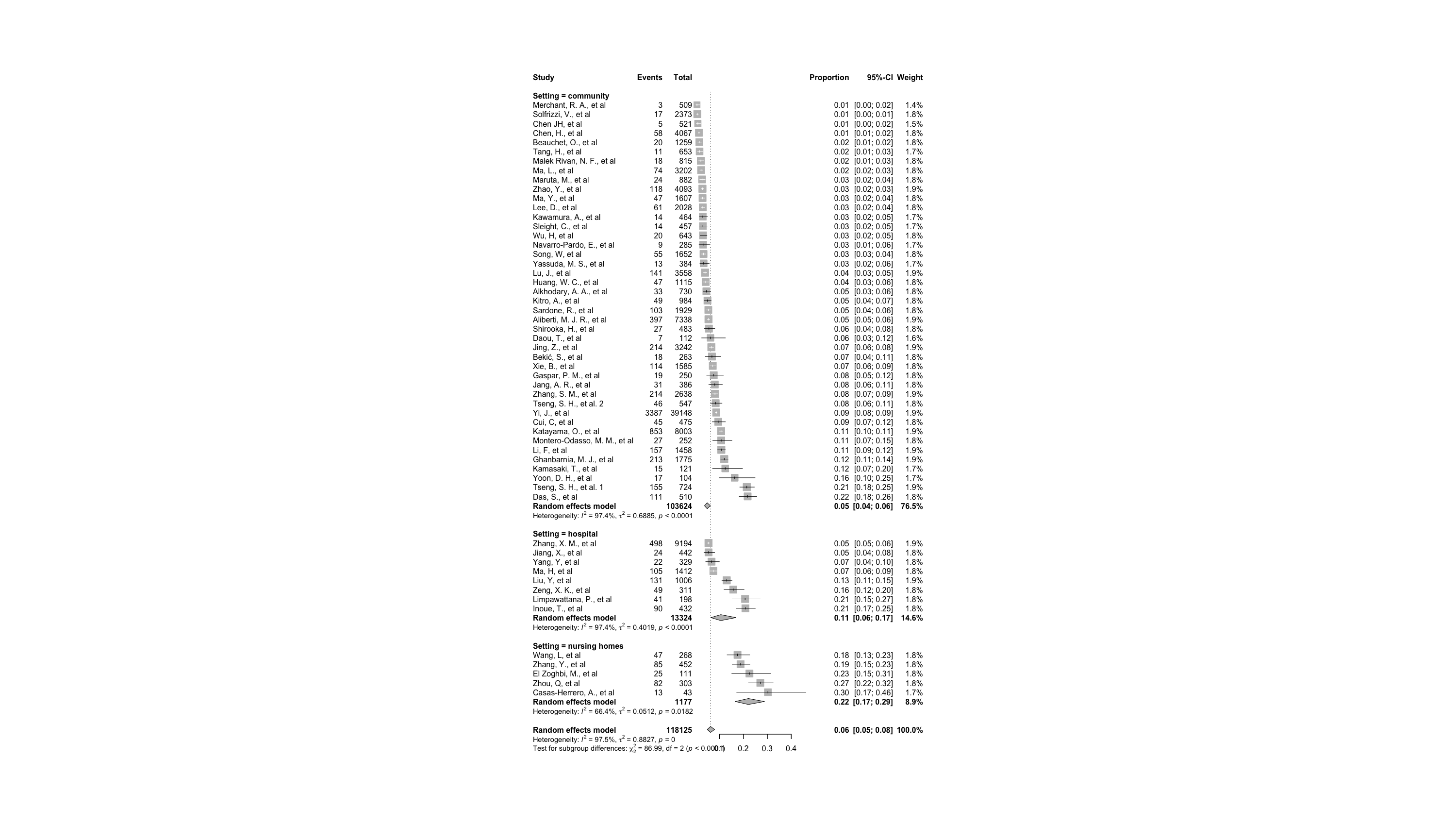


**(B) PRCF**


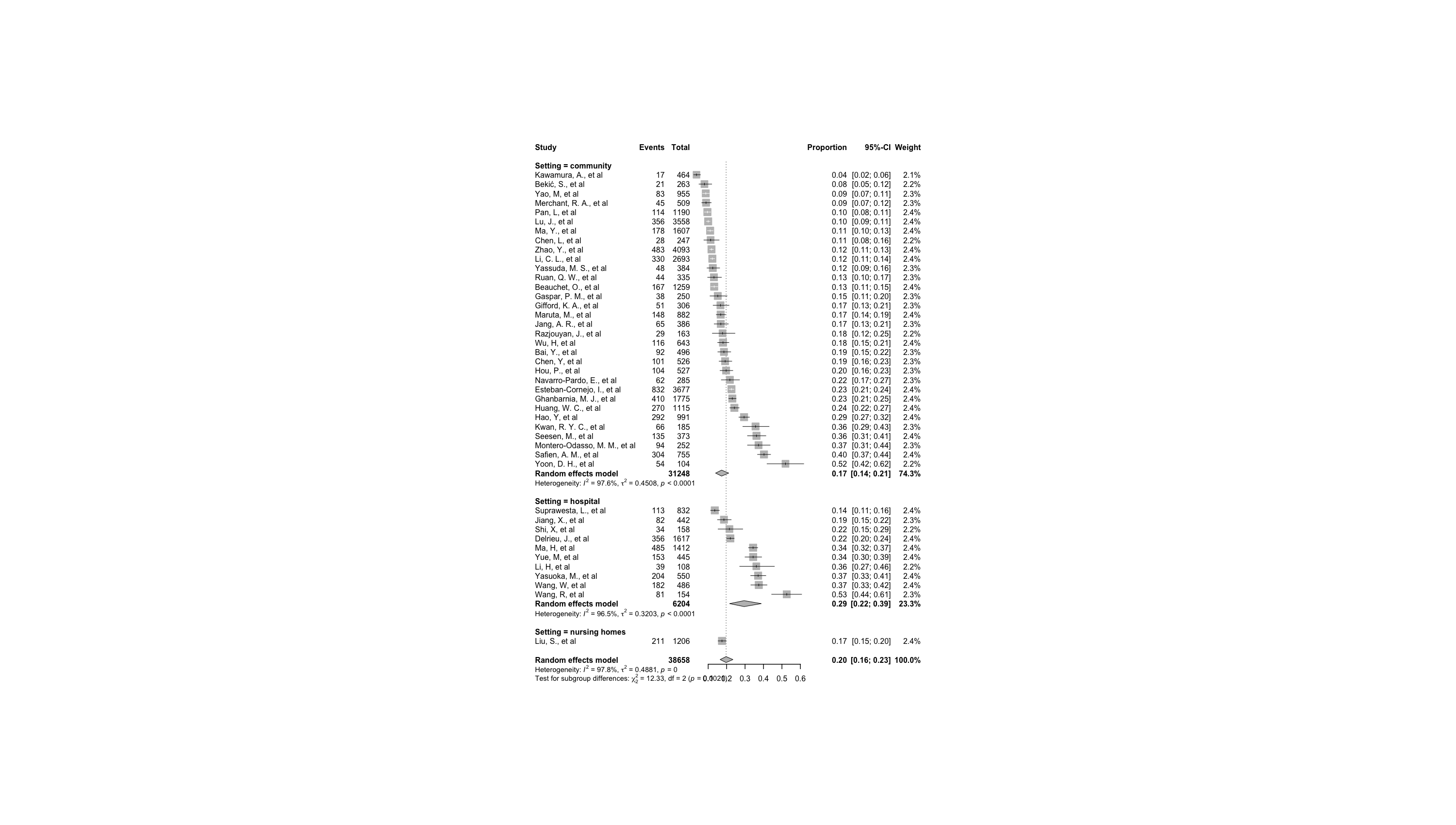


**(C) RCF**


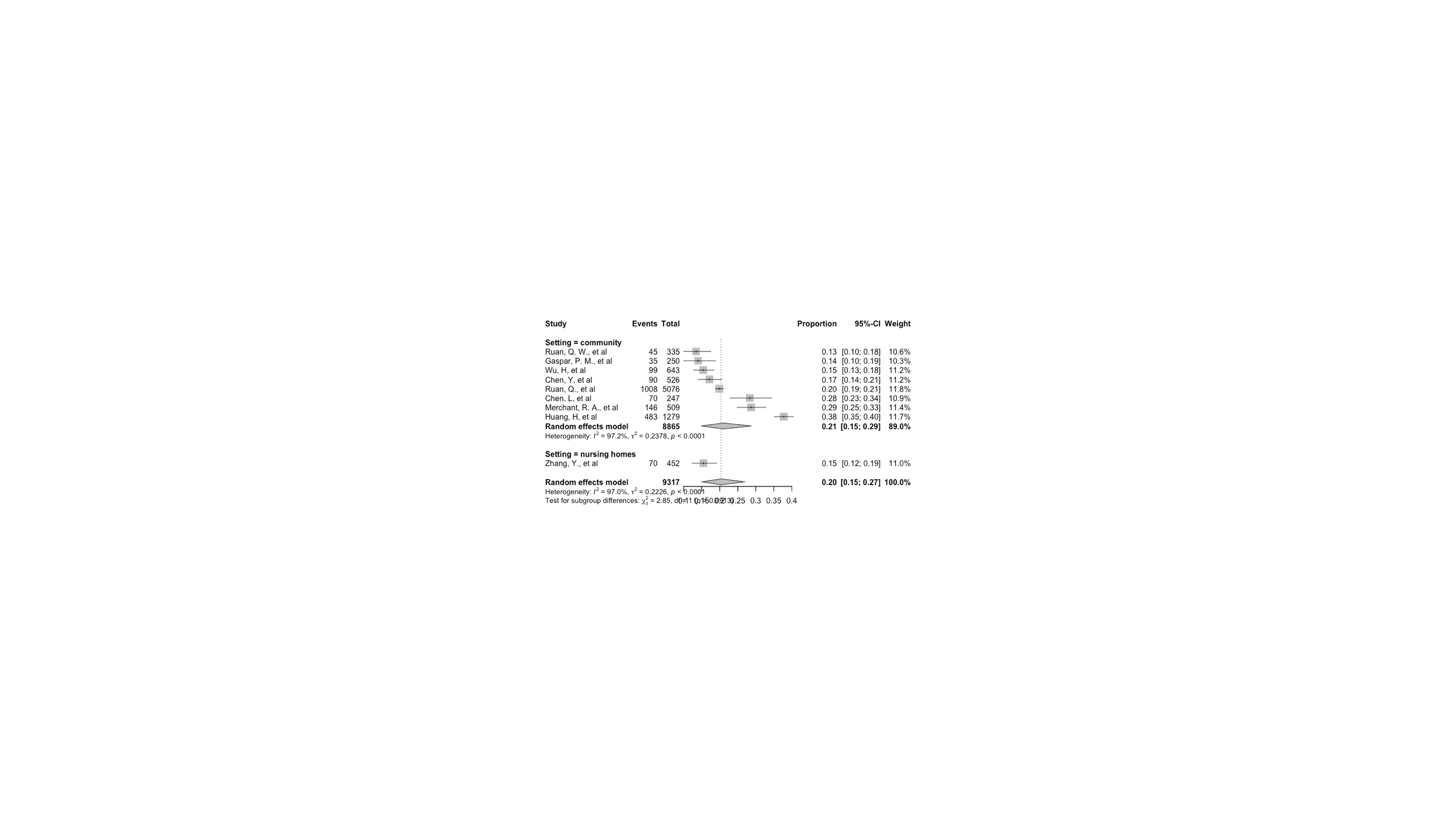


**Supplementary Figure 7.** Prevalence of CF after excluding studies with a high risk of bias

Note: None of the studies on PRCF and RCF were rated as high risk of bias.

Abbreviations: CF: cognitive frailty; PRCF: potentially reversible cognitive frailty; RCF: reversible cognitive frailty


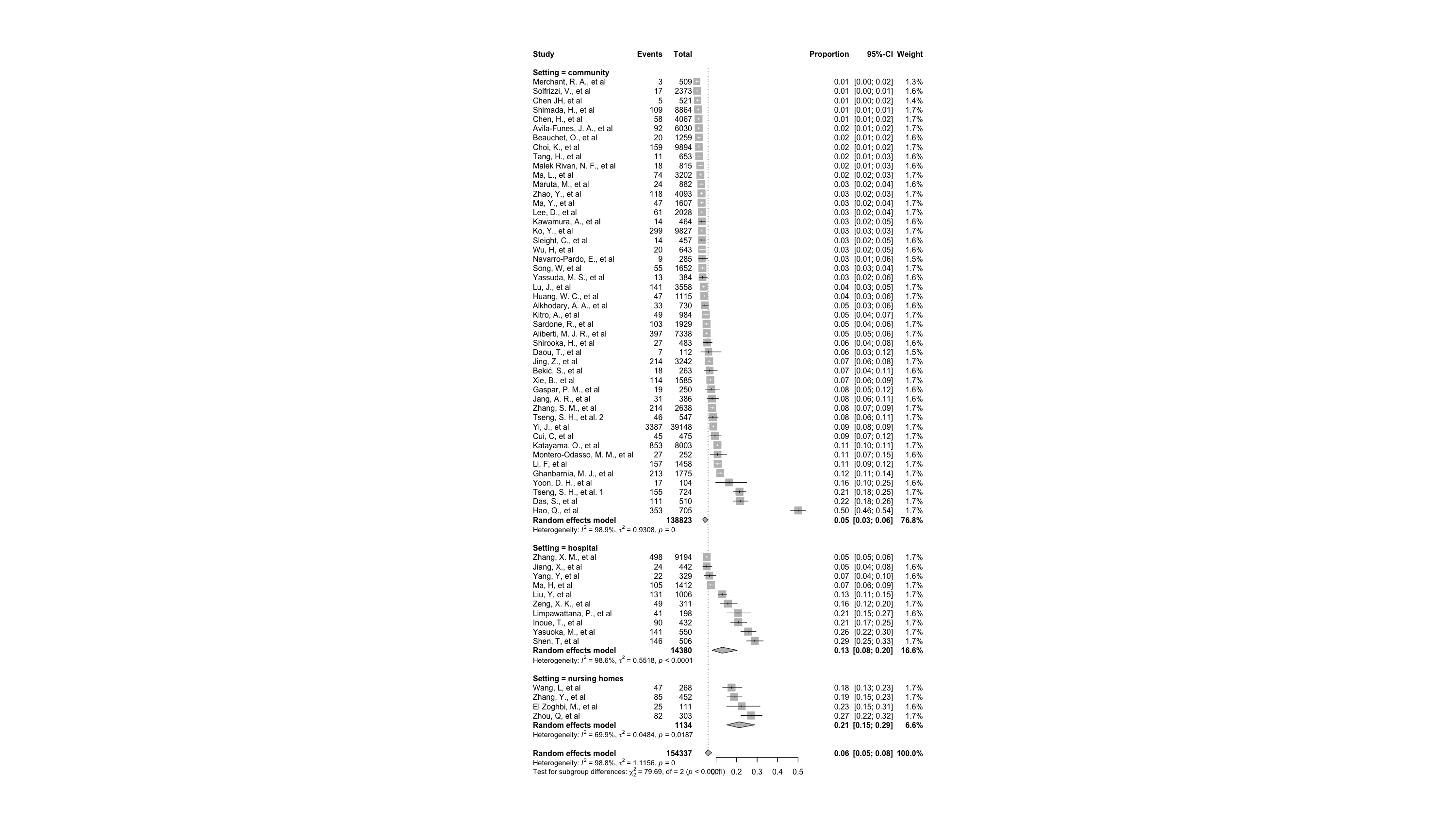


**Supplementary Figure 8.** Funnel plot of the prevalence of (A) CF, (B) PRCF, and (C) RCF

Abbreviations: CF: cognitive frailty; PRCF: potentially reversible cognitive frailty; RCF: reversible cognitive frailty

**(A) CF**

**
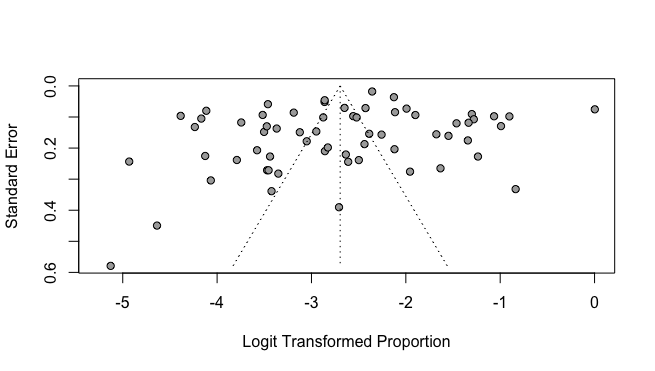
**

**(B) PRCF**

**
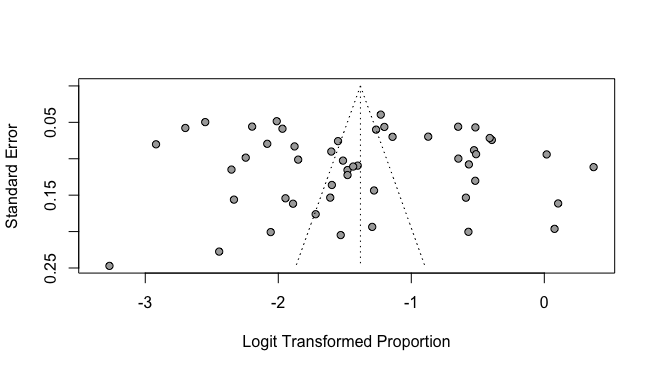
**

**(C) RCF**

**
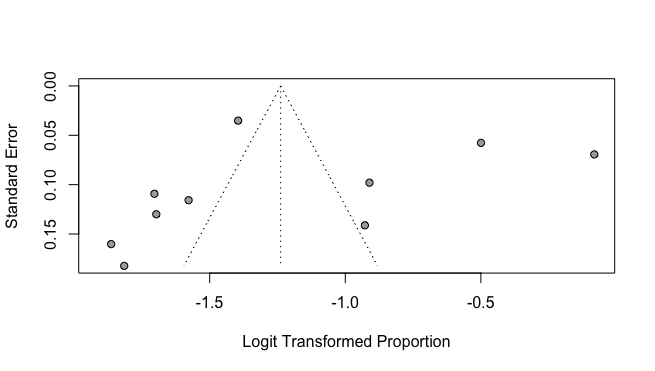
**

**References**

1 Aliberti M J R, I S Cenzer, A K Smith, et al., *Assessing Risk for Adverse Outcomes in Older Adults: The Need to Include Both Physical Frailty and Cognition.* J Am Geriatr Soc, 2019. **67**: 477-483.

2 Alkhodary A A, S M Aljunid, A Ismail, A M Nur, and S Shahar, *Health Care Utilization and Out-of-Pocket Payments among Elderly with Cognitive Frailty in Malaysia.* Int J Environ Res Public Health, 2022. **19**.

3 Avila-Funes J A, H Amieva, P Barberger-Gateau, et al., *Cognitive impairment improves the predictive validity of the phenotype of frailty for adverse health outcomes: the three-city study.* J Am Geriatr Soc, 2009. **57**: 453-61.

4 Bai Y, Y Chen, M Tian, et al., *The Relationship Between Social Isolation and Cognitive Frailty Among Community-Dwelling Older Adults: The Mediating Role of Depressive Symptoms.* Clin Interv Aging, 2024. **19**: 1079-1089.

5 Bekić S, F Babič, V Pavlišková, et al., *Clusters of Physical Frailty and Cognitive Impairment and Their Associated Comorbidities in Older Primary Care Patients.* Healthcare (Basel), 2021. **9**.

6 Beauchet O, J Matskiv, P Gaudreau, et al., *Frailty, Cognitive Impairment, and Incident Major Neurocognitive Disorders: Results of the NuAge Cohort Study.* J Alzheimers Dis, 2023. **94**: 1079-1092.

7 Chen J H, H S Shih, J Tu, et al., *A Longitudinal Study on the Association of Interrelated Factors Among Frailty Dimensions, Cognitive Domains, Cognitive Frailty, and All-Cause Mortality.* J Alzheimers Dis, 2021. **84**: 1795-1809.

8 Casas-Herrero A, E L Cadore, F Zambom-Ferraresi, et al., *Functional capacity, muscle fat infiltration, power output, and cognitive impairment in institutionalized frail oldest old.* Rejuvenation Res, 2013. **16**: 396-403.

9 Chen H, L Huang, W Xiang, Y Liu, and J W Xu, *Association between cognitive frailty and falls among older community dwellers in China: A Chinese longitudinal healthy longevity survey-based study.* Front Aging Neurosci, 2022. **14**: 1048961.

10 Chen L, H Gao, F Wei, and Y Yang, *Correlation analysis on cognitive frailty prevalence and its relationship with expectations regarding aging among the elderly in rural areas of Shanxi Province.* Occup and Health, 2023. **39**: 2944-2948.

11 Chen Y, Z Zhang, Q Zuo, J Liang, and Y Gao, *Construction and validation of a prediction model for the risk of cognitive frailty among the elderly in a community.* Chinese Journal of Nursing, 2022. **57**: 197-203.

12 Choi K and Y Ko, *Cross sectional association between cognitive frailty and disability among community-dwelling older adults: Focus on the role of social factors.* Front Public Health, 2023. **11**: 1048103.

13 Cui C and Q Yang, *Cognitive Frailty of the Elderly in the Community and Its Relationship with Leisure Activities.* Journal of Medical Theory and Practice, 2022. **35**: 377-379+399.

14 Daou T, J Abi Kharma, A Daccache, et al., *Association between Lebanese Mediterranean Diet and Frailty in Community-Dwelling Lebanese Older Adults-A Preliminary Study.* Nutrients, 2022. **14**.

15 Das S, *Cognitive frailty among community-dwelling rural elderly population of West Bengal in India.* Asian J Psychiatr, 2022. **70**: 103025.

16 Delrieu J, S Andrieu, M Pahor, et al., *Neuropsychological Profile of "Cognitive Frailty" Subjects in MAPT Study.* J Prev Alzheimers Dis, 2016. **3**: 151-159.

17 Esteban-Cornejo I, V Cabanas-Sánchez, S Higueras-Fresnillo, et al., *Cognitive Frailty and Mortality in a National Cohort of Older Adults: the Role of Physical Activity.* Mayo Clin Proc, 2019. **94**: 1180-1189.

18 Gaspar P M, M Campos-Magdaleno, A X Pereiro, D Facal, and O Juncos-Rabadán, *Cognitive reserve and mental health in cognitive frailty phenotypes: Insights from a study with a Portuguese sample.* Front Psychol, 2022. **13**: 968343.

19 Ghanbarnia M J, S R Hosseini, A A Ahangar, R Ghadimi, and A Bijani, *Prevalence of cognitive frailty and its associated factors in a population of Iranian older adults.* Aging Clin Exp Res, 2024. **36**: 134.

20 Gifford K A, S P Bell, D Liu, et al., *Frailty Is Related to Subjective Cognitive Decline in Older Women without Dementia.* J Am Geriatr Soc, 2019. **67**: 1803-1811.

21 Hao Q, B Dong, M Yang, B Dong, and Y Wei, *Frailty and Cognitive Impairment in Predicting Mortality Among Oldest-Old People.* Front Aging Neurosci, 2018. **10**: 295.

22 Hao Y and Y Zhao, *Analysis of the Influencing Factors of Cognitive Frailty among Elderly People in the Community Based on Propensity Score Matching.* Nursing, 2024. **13**: 175-180.

23 Hou P, H Xue, Y Zhang, et al., *Mediating Effect of Loneliness in the Relationship between Depressive Symptoms and Cognitive Frailty in Community-Dwelling Older Adults.* Brain Sci, 2022. **12**.

24 Huang H, J Yang, X Yang, et al., *A study on the relationship between reversible cognitive decline and impairment of daily life function, hospitalization, and fall among the elderly in rural areas of Guizhou Province.* Modern Preventive Medicine, 2023. **50**: 3924-3929.

25 Huang W C, Y C Huang, M S Lee, H Y Chang, and J Y Doong, *Frailty Severity and Cognitive Impairment Associated with Dietary Diversity in Older Adults in Taiwan.* Nutrients, 2021. **13**.

26 Inoue T, A Shimizu, S Satake, et al., *Association between osteosarcopenia and cognitive frailty in older outpatients visiting a frailty clinic.* Arch Gerontol Geriatr, 2022. **98**: 104530.

27 Jang A R and J Y Yoon, *Multilevel Factors Associated with Frailty among the Rural Elderly in Korea Based on the Ecological Model.* Int J Environ Res Public Health, 2021. **18**.

28 Jiang X, J Zhou, C Yu, et al., *The alterations in multiple neurophysiological procedures are associated with frailty phenotype in older adults.* Front Aging Neurosci, 2023. **15**: 1063322.

29 Jing Z, J Li, Y Wang, et al., *The mediating effect of psychological distress on cognitive function and physical frailty among the elderly: Evidence from rural Shandong, China.* J Affect Disord, 2020. **268**: 88-94.

30 Kamasaki T, H Otao, M Hachiya, et al., *Social Functioning and Life-Related Domains Associated with Cognitive Frailty in Older Adults.* Physical & Occupational Therapy in Geriatrics, 2023. **41**: 331-346.

31 Katayama O, S Lee, S Bae, et al., *Lifestyle Activity Patterns Related to Physical Frailty and Cognitive Impairment in Urban Community-Dwelling Older Adults in Japan.* J Am Med Dir Assoc, 2021. **22**: 583-589.

32 Kawamura A, N Kamide, M Ando, et al., *The Combination of Hearing Impairment and Frailty Is Associated with Cognitive Decline among Community-Dwelling Elderly in Japan.* Int J Environ Res Public Health, 2023. **20**.

33 Kitro A, J Panumasvivat, W Sirikul, et al., *Associations between frailty and mild cognitive impairment in older adults: Evidence from rural Chiang Mai Province.* PLoS One, 2024. **19**: e0300264.

34 Ko Y and K Choi, *Exploring the Role of Social Factors in Cognitive Frailty among South Korean Older Adults.* Healthcare (Basel), 2024. **12**.

35 Kwan R Y C, A Y M Leung, A Yee, et al., *Cognitive Frailty and Its Association with Nutrition and Depression in Community-Dwelling Older People.* J Nutr Health Aging, 2019. **23**: 943-948.

36 Lee D, M Kim, and C W Won, *Common and different characteristics among combinations of physical frailty and sarcopenia in community-dwelling older adults: The Korean Frailty and Aging Cohort Study.* Geriatr Gerontol Int, 2022. **22**: 42-49.

37 Li C L, H Y Chang, and F F Stanaway, *Combined effects of frailty status and cognitive impairment on health-related quality of life among community dwelling older adults.* Arch Gerontol Geriatr, 2020. **87**: 103999.

38 Li F and Q Hong, *Interaction between vitamin D deficiency and obesity on cognitive frailty among community-dwelling elderly people.* Modern Preventive Medicine, 2021. **48**: 2924-2928.

39 Li H, D Ren, L Yu, et al., *Association between cognitive frailty and sarcopenia in hospitalized elderly population.* Chinese Journal of Practical Internal Medicine, 2024. **44**: 291-295.

40 Limpawattana P, C Khammak, M Manjavong, and A So-Ngern, *Frailty as a Predictor of Hospitalization and Low Quality of Life in Geriatric Patients at an Internal Medicine Outpatient Clinic: A Cross-Sectional Study.* Geriatrics (Basel), 2022. **7**.

41 Liu S, Z Hu, Y Guo, et al., *Association of sleep quality and nap duration with cognitive frailty among older adults living in nursing homes.* Front Public Health, 2022. **10**: 963105.

42 Liu Y, L Yu, T Han, and P Li, *Current situation and influential factors of cognitive frailty among elderly patients in Urumqi, China.* Chinese Journal of Practical Nursing, 2021. **37**: 424-430.

43 Lu J, Q Q Guo, Y Wang, Z X Zuo, and Y Y Li, *The Evolutionary Stage of Cognitive Frailty and Its Changing Characteristics in Old Adults.* J Nutr Health Aging, 2021. **25**: 467-478.

44 Ma H, Y Li, X Li, X Wu, and J Qiao, *Analysis of risk factors and construction of a prediction model for mild cognitive impairment in elderly inpatients in sub-plateau areas.* Chinese Journal of Geriatrics, 2022. **41**: 80-85.

45 Ma L N, L Zhang, F Sun, Y Li, and Z Tang, *Cognitive function in Prefrail and frail community-dwelling older adults in China.* Bmc Geriatrics, 2019. **19**.

46 Ma Y, X Li, Y Pan, et al., *Cognitive frailty predicting death and disability in Chinese elderly.* Neurol Res, 2021. **43**: 815-822.

47 Malek Rivan N F, S Shahar, N F Rajab, et al., *Cognitive frailty among Malaysian older adults: baseline findings from the LRGS TUA cohort study.* Clin Interv Aging, 2019. **14**: 1343-1352.

48 Malek Rivan N F, S Shahar, D K A Singh, et al., *Development of cognitive frailty screening tool among community-dwelling older adults.* Heliyon, 2024. **10**: e34223.

49 Maruta M, S Shimokihara, H Makizako, et al., *Associations between apathy and comprehensive frailty as assessed by the Kihon Checklist among community-dwelling Japanese older adults.* Psychogeriatrics, 2022. **22**: 651-658.

50 Merchant R A, Y H Chan, R J Y Hui, et al., *Motoric cognitive risk syndrome, physio-cognitive decline syndrome, cognitive frailty and reversibility with dual-task exercise.* Exp Gerontol, 2021. **150**: 111362.

51 Montero-Odasso M M, B Barnes, M Speechley, et al., *Disentangling Cognitive-Frailty: Results From the Gait and Brain Study.* J Gerontol A Biol Sci Med Sci, 2016. **71**: 1476-1482.

52 Navarro-Pardo E, D Facal, M Campos-Magdaleno, A X Pereiro, and O Juncos-Rabadán, *Prevalence of Cognitive Frailty, Do Psychosocial-Related Factors Matter?* Brain Sci, 2020. **10**.

53 Pan L, W Zhang, Z Yu, et al., *The current status and influencing factors of cognitive frailty among elderly community residents in Zhengzhou city, China.* Journal of Nursing Science, 2019. **34**: 79-82.

54 Razjouyan J, B Najafi, M Horstman, et al., *Toward Using Wearables to Remotely Monitor Cognitive Frailty in Community-Living Older Adults: An Observational Study.* Sensors (Basel), 2020. **20**.

55 Ruan Q, F Xiao, K Gong, et al., *Prevalence of Cognitive Frailty Phenotypes and Associated Factors in a Community-Dwelling Elderly Population.* J Nutr Health Aging, 2020. **24**: 172-180.

56 Ruan Q W, W B Zhang, J Ruan, J Chen, and Z W Yu, *Clinical and Objective Cognitive Measures for the Diagnosis of Cognitive Frailty Subtypes: A Comparative Study.* Frontiers in Psychology, 2021. **12**.

57 Sardone R, F Castellana, I Bortone, et al., *Association Between Central and Peripheral Age-Related Hearing Loss and Different Frailty Phenotypes in an Older Population in Southern Italy.* JAMA Otolaryngol Head Neck Surg, 2021. **147**: 561-571.

58 Seesen M, W Sirikul, J Ruangsuriya, J Griffiths, and P Siviroj, *Cognitive Frailty in Thai Community-Dwelling Elderly: Prevalence and Its Association with Malnutrition.* Nutrients, 2021. **13**.

59 Shen T and K Zhou, *Correlation Analysis between Cognitive Decline and Oral Health Status in Elderly Patients.* Health vocational education, 2024. **42**: 116-119.

60 Shi X and Q Ma, *Current status and influencing factors of cognitive frailty in the elderly.* Chinese Journal of Multiorgan Diseases of the Elderly, 2022. **21**: 581-586.

61 Shimada H, H Makizako, S Lee, et al., *Impact of Cognitive Frailty on Daily Activities in Older Persons.* J Nutr Health Aging, 2016. **20**: 729-35.

62 Shirooka H, S Nishiguchi, N Fukutani, et al., *Cognitive impairment is associated with the absence of fear of falling in community-dwelling frail older adults.* Geriatr Gerontol Int, 2017. **17**: 232-238.

63 Sleight C and R Holtzer, *Differential associations of functional and cognitive health outcomes with pre-frailty and frailty states in community-dwelling older adults.* Journal of Health Psychology, 2020. **25**: 1057-1063.

64 Solfrizzi V, E Scafato, M Lozupone, et al., *Additive Role of a Potentially Reversible Cognitive Frailty Model and Inflammatory State on the Risk of Disability: The Italian Longitudinal Study on Aging.* Am J Geriatr Psychiatry, 2017. **25**: 1236-1248.

65 Song W, J Yang, X Yang, et al., *Current situation and influencing factors of cognitive frailty in elderly residents of Bouyei nationality area in Guizhou Province.* Chinese Journal of Chronic Disease Prevention and Control, 2021. **29**: 161-166.

66 Sugimoto T, R Ono, A Kimura, et al., *Cross-Sectional Association Between Cognitive Frailty and White Matter Hyperintensity Among Memory Clinic Patients.* J Alzheimers Dis, 2019. **72**: 605-612.

67 Suprawesta L, S J Chen, H Y Liang, et al., *Factors affecting cognitive frailty improvement and progression in Taiwanese older adults.* BMC Geriatr, 2024. **24**: 105.

68 Safien A M, N Ibrahim, P Subramaniam, et al., *Prevalence of depression and clinical depressive symptoms in community-dwelling older adults with cognitive frailty.* Geriatrics & Gerontology International, 2024. **24**: 225-233.

69 Tang H, H Zhu, Q Sun, H Qin, and S Wang, *Transitions in the Cognitive Frailty States in Community-Living Older Adults: A 6-Year Prospective Cohort Study.* Front Aging Neurosci, 2021. **13**: 774268.

70 Tseng S H, L K Liu, L N Peng, et al., *Development and Validation of a Tool to Screen for Cognitive Frailty among Community-Dwelling Elders.* J Nutr Health Aging, 2019. **23**: 904-909.

71 Wang L, C Xu, L Tang, Y Li, and T Rao, *Cognitive frailty and influencing factors among older adults in nursing homes.* Chinese Journal of Gerontology, 2021. **41**: 3554-3557.

72 Wang R, M Li, B Wang, and Y Gao, *Current status and influencing factors of cognitive frailty in hospitalised older adults.* Chinese General Practice Nursing, 2024. **22**: 2540-2543.

73 Wang W, J Kou, T Zhang, and S Yang, *Cognitive frailty of inpatients in Department of Geriatrics and its influencing factors.* Chinese Journal of Modern Nursing, 2022. **28**: 296-301.

74 Wu H, Y Luo, G Yao, et al., *Association between cognitive frailty and self - perceptions of ageing in community-dwelling elderly.* Modern Preventive Medicine, 2021. **48**: 3940-3944+3978.

75 Xie B, C Ma, Y Chen, and J Wang, *Prevalence and risk factors of the co-occurrence of physical frailty and cognitive impairment in Chinese community-dwelling older adults.* Health Soc Care Community, 2021. **29**: 294-303.

76 Yang Y, X Zeng, Q Zhu, and X Chen, *Survey on asthenia status in elderly outpatients and related factors.* Zhejiang Medicine, 2018. **40**: 1050-1053.

77 Yao M, L Meng, C Zhou, et al., *Attribution analysis on the influence of sleep quality on cognitive frailty of community-dwelling elderly people.* Practical Preventive Medicine, 2022. **29**: 1177-1181.

78 Yassuda M S, A Lopes, M Cachioni, et al., *Frailty criteria and cognitive performance are related: data from the FIBRA study in Ermelino Matarazzo, São Paulo, Brazil.* J Nutr Health Aging, 2012. **16**: 55-61.

79 Yasuoka M, M Shinozaki, K Kinoshita, et al., *Prediction of Nursing Home Admission Using the FRAIL-NH Scale Among Older Adults in Post-Acute Care Settings.* Journal of Nutrition Health & Aging, 2023. **27**: 213-218.

80 Yi J and J Y Yoon, *Cognitive frailty increases the risk of long-term care dependency in community-dwelling older adults: A nationwide cohort study in South Korea.* Geriatr Gerontol Int, 2023. **23**: 117-123.

81 Yoon D H, S S Hwang, D W Lee, C G Lee, and W Song, *Physical Frailty and Cognitive Functioning in Korea Rural Community-Dwelling Older Adults.* J Clin Med, 2018. **7**.

82 Yue M, J Kou, F Zhou, et al., *Study on the current status and influencing factors of cognitive frailty in older inpatients of internal medicine department.* Chinese Journal for Clinicians, 2022. **50**: 1051-1054.

83 Zeng X K, S S Shen, H L Guan, L Y Chen, and X J Chen, *Coexisting Frailty and Cognitive Impairment as a Predictor of Adverse Outcomes in Older Inpatients After Discharge: Results from a One-Year Follow-Up Study.* Clin Interv Aging, 2022. **17**: 1697-1706.

84 Zhang S, Q Wang, X Wang, et al., *Pet ownership and cognitive frailty among Chinese rural older adults who experienced a social loss: Is there a sex difference?* Soc Sci Med, 2022. **305**: 115100.

85 Zhang X M, J Jiao, N Guo, et al., *The association between cognitive impairment and 30-day mortality among older Chinese inpatients.* Front Med (Lausanne), 2022. **9**: 896481.

86 Zhang Y, M R Li, X Chen, et al., *Prevalence and risk factors of cognitive frailty among pre-frail and frail older adults in nursing homes.* Psychogeriatrics, 2024. **24**: 529-541.

87 Zhao Y, Y Lu, W Zhao, et al., *Long sleep duration is associated with cognitive frailty among older community-dwelling adults: results from West China Health and Aging Trend study.* BMC Geriatr, 2021. **21**: 608.

88 Zhou Q, J Zhou, M Ku, H Wu, and S Xie, *The prevalence and determinants of cognitive frailty among institutionalized older adults.* Journal of Nursing Science, 2020. **35**: 88-92.

89 El Zoghbi M, C Boulos, A H Amal, et al., *Association between cognitive function and nutritional status in elderly: A cross-sectional study in three institutions of Beirut-Lebanon.* Geriatric Mental Health Care, 2013. **1**: 73-81.

90 Fried L P, C M Tangen, J Walston, et al., *Frailty in older adults: evidence for a phenotype.* J Gerontol A Biol Sci Med Sci, 2001. **56**: M146-56.

91 Morley J E, B Vellas, G A van Kan, et al., *Frailty consensus: a call to action.* J Am Med Dir Assoc, 2013. **14**: 392-7.

92 Studenski S, S Perera, K Patel, et al., *Gait speed and survival in older adults.* Jama, 2011. **305**: 50-8.

93 Rockwood K and A Mitnitski, *Frailty in relation to the accumulation of deficits.* J Gerontol A Biol Sci Med Sci, 2007. **62**: 722-7.

94 Rockwood K, X Song, C MacKnight, et al., *A global clinical measure of fitness and frailty in elderly people.* Cmaj, 2005. **173**: 489-95.

95 *Japanese Ministry of Health, Labour and Welfare. The Manuals of the Evaluation for Ability to Perform Daily Activities on Preventive Care.*; Available from: <http://www.mhlw.go.jp/topics/2009/05/dl/tp0501-1c_0001.pdf>.

96 Ensrud K E, S K Ewing, B C Taylor, et al., *Comparison of 2 frailty indexes for prediction of falls, disability, fractures, and death in older women.* Arch Intern Med, 2008. **168**: 382-9.

97 Podsiadlo D and S Richardson, *The timed "Up & Go": a test of basic functional mobility for frail elderly persons.* J Am Geriatr Soc, 1991. **39**: 142-8.

98 Gobbens R J, M A van Assen, K G Luijkx, M T Wijnen-Sponselee, and J M Schols, *The Tilburg Frailty Indicator: psychometric properties.* J Am Med Dir Assoc, 2010. **11**: 344-55.

99 Folstein M F, S E Folstein, and P R McHugh, *"Mini-mental state". A practical method for grading the cognitive state of patients for the clinician.* J Psychiatr Res, 1975. **12**: 189-98.

100 Nasreddine Z S, N A Phillips, V Bédirian, et al., *The Montreal Cognitive Assessment, MoCA: a brief screening tool for mild cognitive impairment.* J Am Geriatr Soc, 2005. **53**: 695-9.

101 Morris J C, *The Clinical Dementia Rating (CDR): current version and scoring rules.* Neurology, 1993. **43**: 2412-4.

102 Makizako H, H Shimada, H Park, et al., *Evaluation of multidimensional neurocognitive function using a tablet personal computer: test-retest reliability and validity in community-dwelling older adults.* Geriatr Gerontol Int, 2013. **13**: 860-6.

103 Borson S, J Scanlan, M Brush, P Vitaliano, and A Dokmak, *The mini-cog: a cognitive 'vital signs' measure for dementia screening in multi-lingual elderly.* Int J Geriatr Psychiatry, 2000. **15**: 1021-7.

104 Storey J E, J T Rowland, D Basic, D A Conforti, and H G Dickson, *The Rowland Universal Dementia Assessment Scale (RUDAS): a multicultural cognitive assessment scale.* Int Psychogeriatr, 2004. **16**: 13-31.

105 Galvin J E, C M Roe, K K Powlishta, et al., *The AD8: a brief informant interview to detect dementia.* Neurology, 2005. **65**: 559-64.

106 Imai Y and K Hasegawa, *The Revised Hasegawa's Dementia Scale (HDS-R)-Evaluation of Its Usefulness as a Screening Test for Dementia.* Hong Kong Journal of Psychiatry, 1994. **4**: 20.

107 Yang D-W, J-Y CHEY, S-Y KIM, and B-S KIM, *The development and validation of Korean dementia screening questionnaire (KDSQ).* Journal of the Korean Neurological Association, 2002: 135-141.

108 Randolph C, M C Tierney, E Mohr, and T N Chase, *The Repeatable Battery for the Assessment of Neuropsychological Status (RBANS): preliminary clinical validity.* J Clin Exp Neuropsychol, 1998. **20**: 310-9.

109 Malmstrom T K, V B Voss, D M Cruz-Oliver, et al., *The Rapid Cognitive Screen (RCS): A Point-of-Care Screening for Dementia and Mild Cognitive Impairment.* J Nutr Health Aging, 2015. **19**: 741-4.

110 Ura C, F Miyamae, N Sakuma, et al., *[Development of a self-administered dementia checklist (SDC) (1): Examination of factorial validity and internal reliability].* Nihon Ronen Igakkai Zasshi, 2015. **52**: 243-53.

111 Pfeiffer E, *A short portable mental status questionnaire for the assessment of organic brain deficit in elderly patients.* J Am Geriatr Soc, 1975. **23**: 433-41.

112 Teng E L and H C Chui, *The Modified Mini-Mental State (3MS) examination.* J Clin Psychiatry, 1987. **48**: 314-8.

113 Jonker C, L J Launer, C Hooijer, and J Lindeboom, *Memory complaints and memory impairment in older individuals.* J Am Geriatr Soc, 1996. **44**: 44-9.

114 Jessen F, R E Amariglio, M van Boxtel, et al., *A conceptual framework for research on subjective cognitive decline in preclinical Alzheimer's disease.* Alzheimers Dement, 2014. **10**: 844-52.

115 Royle J and N B Lincoln, *The Everyday Memory Questionnaire-revised: development of a 13-item scale.* Disabil Rehabil, 2008. **30**: 114-21.

116 Rami L, M A Mollica, C García-Sanchez, et al., *The Subjective Cognitive Decline Questionnaire (SCD-Q): a validation study.* J Alzheimers Dis, 2014. **41**: 453-66.
